# Impact of clinical case discussions in anatomy on national exam performance: a 10-year longitudinal cohort study

**DOI:** 10.1186/s12909-026-10288-6

**Published:** 2026-08-27

**Authors:** Fang Fang, Lan Yu, Yetao Ou, Si Zhou, Jun Ma, Yuntao Peng, Fang Liu, Junfeng Wang, Chunbo Xia, Houzhong Li, Weishan Zhang, Tan Cheng, Yu Chen, Yi Huang, Xin Li, Heng Guan

**Affiliations:** Department of Human Anatomy, Guilin Medical University, No. 1, Zhiyuan Road, Lingui District, Guilin, Guangxi Zhuang Autonomous Region 541199 China

**Keywords:** Case-based learning, Curriculum reform, Longitudinal cohort, Systematic anatomy, National standardized examination

## Abstract

**Background:**

Traditional anatomy instruction often prioritizes structural memorization over clinical application. Although case-based learning is widely endorsed to bridge this theory-practice gap, longitudinal evidence regarding its sustained impact in foundational sciences remains limited, particularly concerning its performance over a decade amid massive enrollment expansion.

**Methods:**

This 10-year longitudinal retrospective cohort study tracked seven clinical medicine cohorts (2015-2021). To maintain teaching quality amid a massive enrollment expansion initiated in 2018, cohorts 2015-2017 received traditional instruction (control), while 2018-2021 underwent structured clinical case discussions (experimental). Primary outcomes were anatomy mastery rate differentials (university performance minus national benchmarks) from two national examinations: the Proficiency Test (stratified into integrated and non-integrated questions) and the National Medical Licensing Examination (overall mastery).

**Results:**

In the Proficiency Test, the experimental group outperformed the national average in integrated questions, peaking at +3.37 (2018 cohort), while non-integrated differentials reached +5.91 (2020 cohort). However, the 2021 cohort's integrated differential sharply declined to -5.34, coinciding temporally with the stagnation of the case repository (the case scenarios had remained unchanged since the 2020 cohort). In the National Medical Licensing Examination, the experimental group's overall differential peaked at +5.69 (2019 cohort). Despite enrollment expanding from 408 to a peak of 795 students, performance remained stable across both examinations.

**Conclusions:**

The implementation of clinical case discussions was associated with improved performance on integrated clinical-anatomy questions in national examinations, and performance remained stable despite substantial enrollment expansion. The observed decline in the 2021 cohort coincided with stagnation of the case repository, suggesting that periodic content renewal may be important for sustaining pedagogical effectiveness. These findings highlight the need for future research on dynamic case repositories and adaptive assessment strategies in the context of emerging educational technologies.

**Supplementary Information:**

The online version contains supplementary material available at https://doi.org/10.1186/s12909-026-10288-6.

## Background

Traditional anatomy instruction in early medical curricula often prioritizes structural memorization over clinical application, widening the theory-practice gap and impeding early clinical reasoning development [1, 2]. Concurrently, China’s direct undergraduate-entry medical education model has undergone rapid enrollment expansion to meet national healthcare demands, creating a dual challenge: bridging foundational sciences with clinical reasoning while maintaining pedagogical fidelity in large-cohort settings [3, 4].

While case-based learning (CBL) is widely endorsed in clinical and paraclinical training to address these challenges, a summary of the existing literature reveals that longitudinal evidence regarding its sustained impact on foundational science assessments remains limited. Existing studies predominantly report short-term outcomes or focus on small-scale implementations, leaving a critical knowledge gap regarding how case-based pedagogy performs over a decade under real-world pressures such as enrollment scaling, content stagnation, and emerging technological disruptions [5, 6].

This study was necessary to bridge this gap and provide empirical evidence for sustaining educational quality during massive cohort expansion. Therefore, the aim of this 10-year longitudinal cohort study was to evaluate the long-term association between the integration of structured clinical case discussions into undergraduate Systematic Anatomy and national standardized examination performance. The primary objective was to compare anatomy mastery rate differentials between traditional lecture-based instruction (cohorts 2015–2017) and case discussion interventions (cohorts 2018–2021) across two national examinations, with a secondary objective to identify implementation factors influencing pedagogical sustainability. We hypothesized that cohorts receiving structured clinical case discussions would demonstrate improved performance on integrated clinical-anatomy questions compared with cohorts receiving traditional lecture-based instruction.

## Methods

### Ethical considerations

This retrospective study utilized fully anonymized national examination records. As no personal identifiers were collected and the research involved minimal risk, the requirement for informed consent was waived. The study protocol was reviewed and approved by the Medical Ethics Committee of Guilin Medical University (Acceptance Number: 202607061101). All data handling adhered to institutional privacy standards.

### Study design, setting, and aim

This 10-year longitudinal retrospective cohort study was conducted at Guilin Medical University, a medical university in China. The primary aim of the study was to evaluate the long-term association between the integration of structured clinical case discussions into undergraduate Systematic Anatomy and students’ performance in national standardized examinations.

### Participants

The study population comprised seven consecutive admission cohorts (2015–2021) of undergraduate students enrolled in the clinical medicine program. Baseline characteristics, including age distribution and national college entrance examination scores, were comparable across the cohorts. All formally enrolled students were included in the cohort tracking. Participants were excluded from specific national assessment cycles only if they were absent due to academic non-completion, post-graduation career transitions away from clinical medicine, or failure to pass the mandatory practical skills prerequisite for the written examination.

### Interventions and comparisons

In China, undergraduate medical education follows a direct-entry model in which students enroll in a five-year clinical medicine program immediately after graduating from senior high school via the National College Entrance Examination (Gaokao). Two national standardized assessments serve as key benchmarks for evaluating medical education quality. Both examinations are developed and administered by the National Medical Examination Center, a statutory body directly under the National Health Commission of China and one of the earliest national professional examination agencies established in the country. The Clinical Medicine Major (Undergraduate) Proficiency Test, co-administered with the National Medical Education Development Center, is conducted for fourth-year students prior to the commencement of clinical clerkship. Students who fail the examination are required to take a make-up examination or retake the assessment; failure to ultimately pass may affect graduation. Examination items are categorized as integrated (requiring synthesis of basic and clinical knowledge) or non-integrated (assessing discrete factual recall). The National Medical Licensing Examination is a high-stakes post-graduation examination administered after one year of supervised clinical practice; passing this examination is a legal prerequisite for independent medical practice, with no limit on the number of attempts. Both examinations use nationally unified test items and standardized scoring criteria, enabling cross-institutional comparability. Following each examination cycle, the National Medical Examination Center provides participating institutions with an official score analysis report for their graduating cohort, from which institutional mastery rates and national benchmarks are derived.

Based on the pedagogical intervention, the cohorts were stratified into two groups for comparison. The control group (cohorts 2015–2017) received traditional lecture-based anatomy instruction. The experimental group (cohorts 2018–2021) participated in structured clinical case discussions integrated into laboratory sessions. The instructional materials consisted of standardized clinical case scenarios co-developed by clinical physicians and anatomy faculty. These discussions were systematically implemented following the completion of core anatomical units, sequentially covering the locomotor system, splanchnology, vascular system, special sense organs, and nervous systems.

The primary outcomes were anatomy mastery rate differentials (calculated as the university’s performance minus the officially published national averages) from two national assessments. To ensure data authority and reliability, the performance data and national benchmarks were derived from official score analysis reports provided by authoritative national bodies: the National Medical Examination Center and the National Medical Education Development Center for the fourth-year Clinical Medicine Major (Undergraduate) Proficiency Test (cohorts 2017–2021), and the National Medical Examination Center for the post-graduation National Medical Licensing Examination (cohorts 2015–2019). For the Proficiency Test, examination items were stratified into integrated (clinical-anatomy) and non-integrated (discrete factual) categories. The Licensing Examination was evaluated based on overall anatomy mastery.

### Statistics

Examination data were analyzed using descriptive statistics. Because the study involved the entire population of enrolled cohorts (census data) rather than a random sample, inferential statistical testing and a priori power calculations were not applicable. The primary outcome metric was the mean anatomy mastery rate differential, calculated for each cohort. Longitudinal trends in these differentials were plotted and compared between the traditional teaching group and the clinical case discussion group to evaluate academic performance trajectories over the study period. Data organization and visualization were performed using Microsoft Excel 2019.

## Results

### Clinical medicine major (undergraduate) proficiency test

**Fig. 1:**
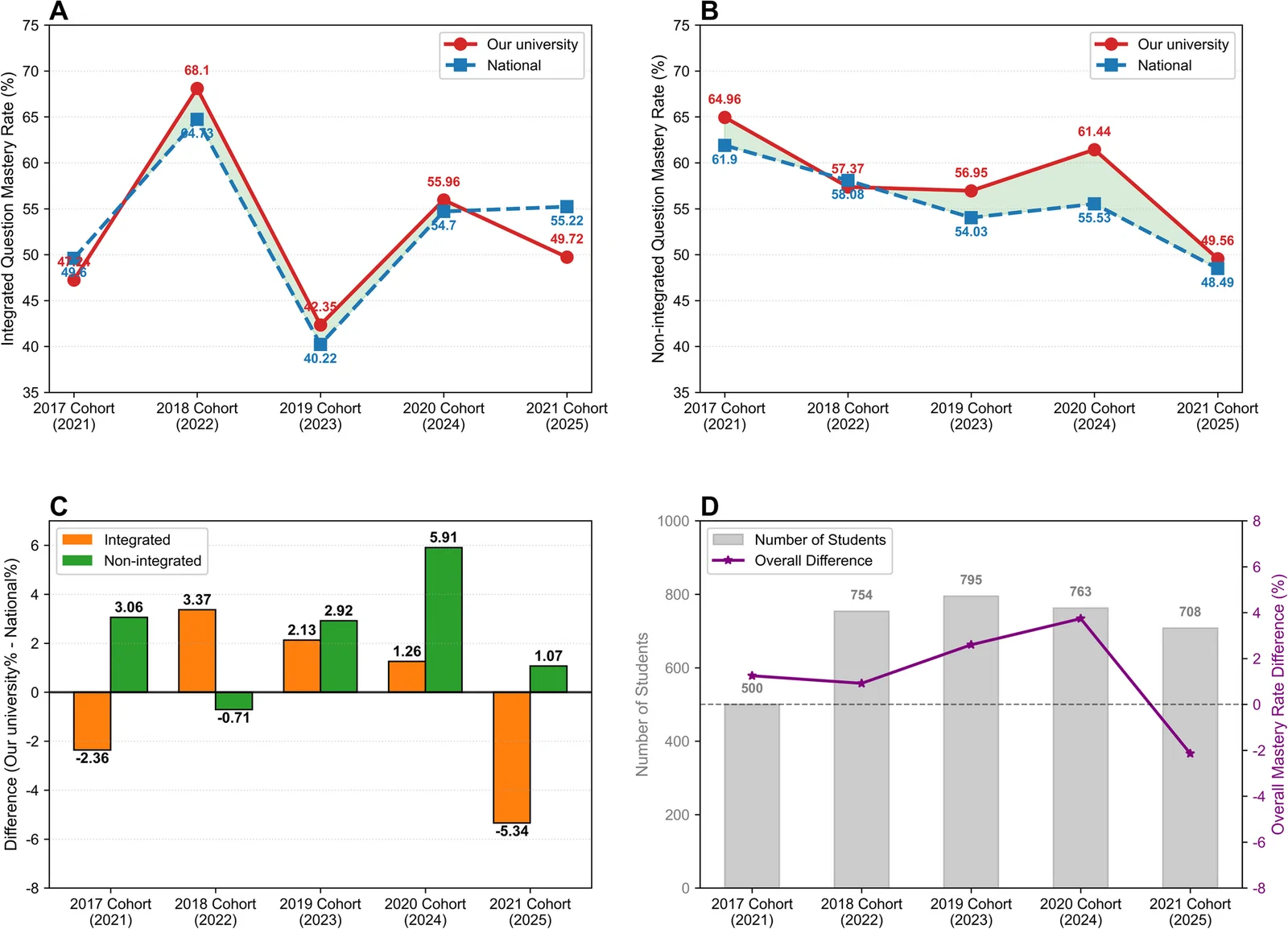
Analysis of anatomy mastery rates in the clinical medicine major (undergraduate) proficiency test. **A **Comparison of Mastery Rates for Integrated Questions; (**B**) Comparison of Mastery Rates for Non-Integrated Questions; (**C**) Discrepancy Analysis of Our University Relative to the National Average; (**D**) Trends in Candidate Numbers and Overall Mastery Rates at Our University

The anatomy performance trends in the Proficiency Test (cohorts 2017–2021) are summarized in Fig. 1. Regarding the discrepancy relative to the national average (Fig. 1C), the traditional teaching group (2017 cohort) fell below the national average for integrated questions, with a mastery rate differential of − 2.36. Following the implementation of case discussions, the experimental group consistently outperformed the national benchmark, peaking at + 3.37 in the 2018 cohort. However, this upward trajectory reversed in the 2021 cohort, which exhibited a substantial decline to a differential of -5.34. This decline coincided temporally with the stagnation of the case repository (the case scenarios had remained unchanged since the 2020 cohort). For non-integrated questions (Fig. 1C), the 2017 traditional cohort demonstrated a differential of + 3.06. The experimental cohorts showed fluctuating but generally positive differentials, reaching a peak of + 5.91 in the 2020 cohort, before returning to a marginal positive differential of + 1.07 in the 2021 cohort.

To further illustrate the absolute performance, the mastery rates for integrated and non-integrated questions are depicted in Fig. 1A and B, respectively. Notably, the overall institutional anatomy mastery rates closely reflected these trajectories (Fig. 1D). The 2020 cohort achieved an overall mastery rate of 58.88% (integrated: 55.96%; non-integrated: 61.44%), whereas the 2021 cohort exhibited a sharp drop to an overall mastery rate of 49.72% (integrated: 49.88%; non-integrated: 49.56%). The concomitant longitudinal trends in candidate numbers are also detailed in Fig. 1D.

### National Medical Licensing Examination

**Fig. 2:**
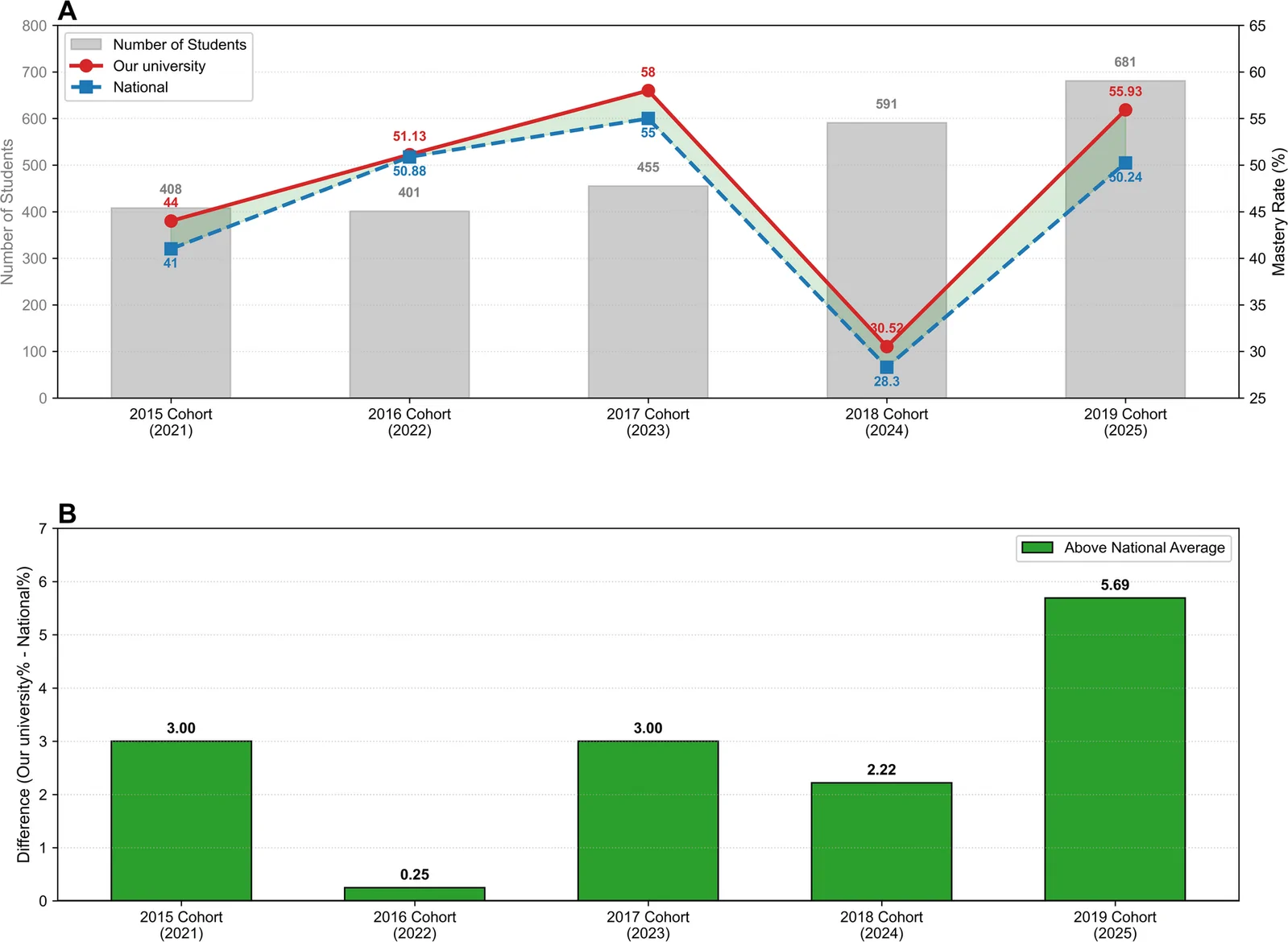
Analysis of anatomy mastery rates in the National Medical Licensing Examination. **A **Comparison of Candidate Numbers and Mastery Rates; (**B**) Discrepancy Analysis of Our University Relative to the National Average

The anatomy performance in the Licensing Examination (cohorts 2015–2019) is detailed in Fig. 2. Regarding the discrepancy relative to the national average (Fig. 2B), the traditional teaching group (cohorts 2015–2017) consistently exceeded the national average, with differentials ranging from + 0.25 to + 3.00, albeit with observable cohort-to-cohort fluctuations. In contrast, the case discussion group (cohorts 2018–2019) demonstrated a discernible upward trajectory. The overall differential increased from + 2.22 in the 2018 cohort to a peak of + 5.69 in the 2019 cohort, representing the highest performance recorded during the study period, suggesting improved overall anatomy mastery in the experimental cohorts. The corresponding trends in candidate numbers and absolute mastery rates are illustrated in Fig. 2A.

### Comparative analysis and enrollment trends

**Fig. 3:**
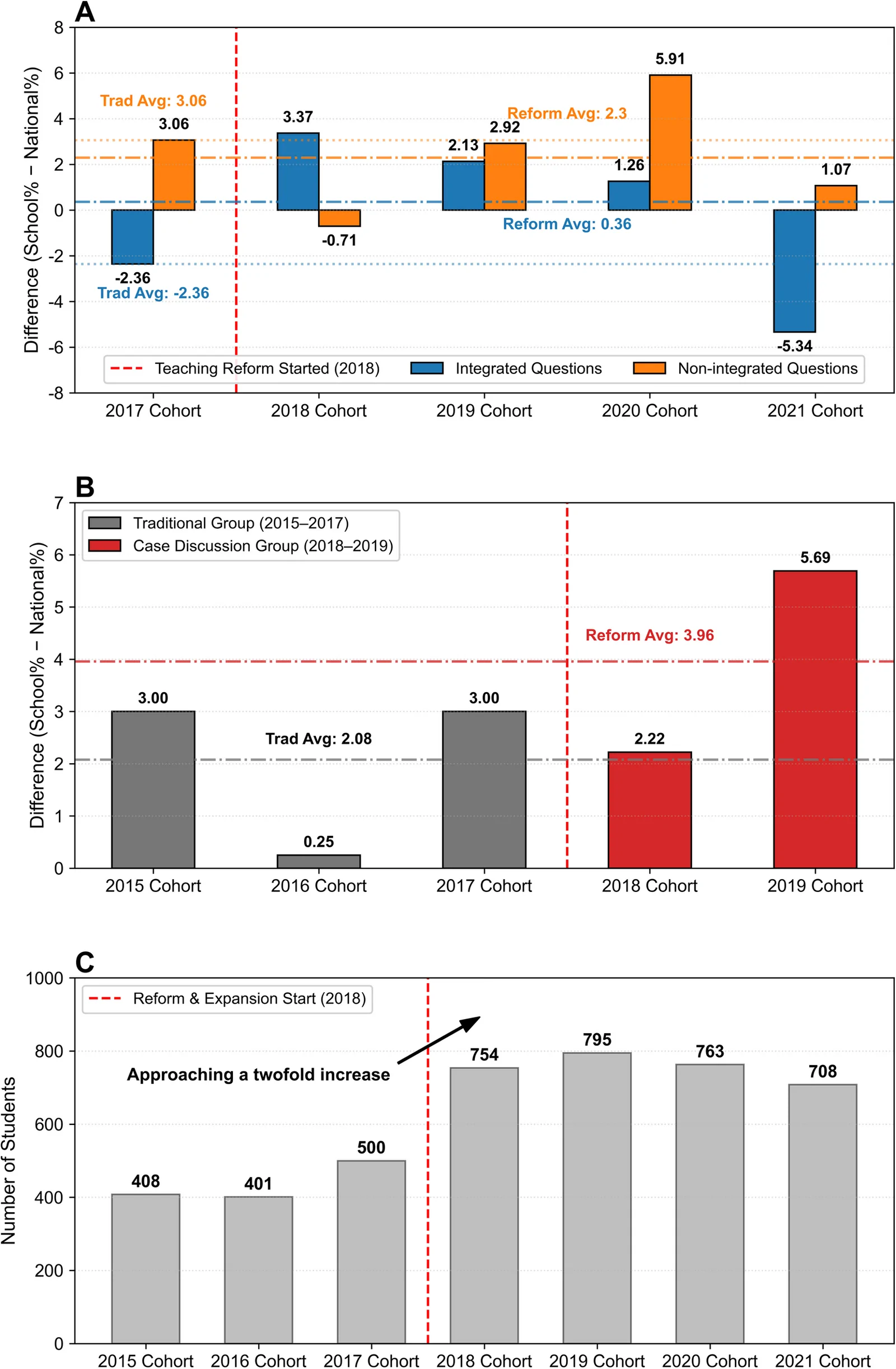
Longitudinal assessment trends in systematic anatomy learning outcomes across clinical medicine cohorts. **A **Temporal trajectories of overall anatomy mastery rates by cohort in the Clinical Medicine Major (Undergraduate) Proficiency Test; (**B**) Mean score differentials on integrated clinical-anatomy questions in the National Medical Licensing Examination: control vs. experimental groups; (**C**) Enrollment trajectory of clinical medicine undergraduate cohorts (2015–2021)

To comprehensively evaluate the teaching reform, comparative analyses of overall performance trajectories and mean differentials were conducted (Fig. 3). In the Proficiency Test, the temporal trajectory of the overall anatomy mastery rate (Fig. 3A) indicated that the average differential for integrated questions shifted from a negative value (− 2.36) in the traditional group to a positive value (+ 0.36) in the experimental group, while the average differential for non-integrated questions decreased from + 3.06 to + 2.30. In the Licensing Examination, the mean score differential on integrated clinical-anatomy questions (Fig. 3B) increased from + 2.08 in the traditional group to + 3.96 in the experimental group.

Regarding enrollment trends, the clinical medicine cohort size exhibited a general upward trajectory (Fig. 3C), expanding from 408 students in the 2015 cohort to a peak of 795 students in the 2019 cohort, before settling at 708 students in the 2021 cohort. The 2018 cohort marked both the initiation of this substantial expansion and the implementation of the teaching reform. Despite the overall increase in student numbers, the experimental group maintained stable or improved performance across multiple assessment indicators. This pattern suggests that the implementation of clinical case discussions was associated with stable performance despite large-scale enrollment expansion.

## Discussion

This 10-year longitudinal cohort study found that the implementation of structured CBL in Systematic Anatomy instruction was associated with improved performance on integrated, clinically contextualized questions, while performance on discrete factual recall remained relatively stable or showed a slight decline. The observed gains in integrated question performance are consistent with established educational theories suggesting that embedding foundational knowledge within authentic clinical scenarios may strengthen cognitive scaffolding for diagnostic reasoning and facilitate the transition from rote memorization to applied synthesis [1, 5, 7]. However, our findings contrast with studies reporting uniform improvements across all assessment types; the reallocation of cognitive resources and instructional time toward higher-order clinical reasoning may temporarily reduce the focus on isolated factual memorization, highlighting the potential trade-offs in curriculum redesign [1, 7].

An important observation from this study is that performance remained stable or improved despite near-doubling of cohort size following the introduction of CBL. While the mere adoption of active learning formats is often championed, our findings suggest that the structured integration of CBL may have contributed to offsetting the pedagogical challenges typically associated with massive enrollment expansion. Specifically, the maintenance of manageable instructor-to-student ratios through multi-session small-class models, coupled with calibrated time controls and uniform scoring rubrics, may have helped preserve active learning integrity [4, 8]. However, this interpretation remains speculative, as the study design cannot isolate the specific contribution of these implementation factors from other concurrent changes.

Furthermore, the longitudinal tracking revealed a notable decline in integrated performance during the 2021 cohort. One possible explanation for this decline is the stagnation of the case repository. Because the materials remained unchanged from 2020 onwards, it is conceivable that some students in the 2021 cohort had access to fixed content and may have replicated the answers of previous senior students, thereby bypassing independent inquiry [4]. However, this explanation remains hypothetical, as no direct evidence (e.g., plagiarism detection data, student surveys, or qualitative feedback) was collected to confirm this mechanism. Faculty composition remained stable throughout the study period, with each class consistently staffed by two instructors (at least one of whom was experienced) and with standardized lesson preparation protocols maintained across all sessions. Therefore, other factors, such as cohort-specific differences in academic preparation, variations in student learning behaviors, or external disruptions, may also have contributed to the observed decline.

Looking ahead, subsequent cohorts (from 2022 onwards) may face additional challenges related to the widespread adoption of generative artificial intelligence (AI), which has the potential to further facilitate academic shortcutting in case-based assessments [4]. Notably, faculty members have anecdotally observed, since the 2023 cohort, a gradual increase in students submitting AI-generated responses during case discussions; however, this observation has not been systematically documented or formally assessed. We therefore raise this issue not as a finding of the current study, but as a future consideration that warrants attention. As the existing evidence on CBL efficacy was predominantly established prior to the widespread availability of generative AI, the interaction between emerging AI tools and case-based pedagogy remains underexplored. Future research should investigate this interaction and explore strategies for maintaining authentic cognitive engagement in this evolving landscape.

Several limitations of this study should be acknowledged. First, the single-institution retrospective design precludes causal inference and restricts generalizability. Because the intervention and control groups consisted of different cohorts assessed in different years, the observed differences in performance cannot be attributed solely to the pedagogical intervention. Unmeasured confounders (such as inter-cohort variations in baseline academic preparation, concurrent curricular modifications outside the anatomy course, and external factors like public health events or policy changes during the study period) may have influenced the results. Second, the primary reliance on high-stakes standardized test scores, while objective and nationally benchmarked, may not fully capture multidimensional clinical reasoning competencies or non-cognitive skills [9, 10]. Third, the observation window for national examination data is truncated at the 2021 cohort (for the Proficiency Test) and 2019 cohort (for the Licensing Exam). Because subsequent cohorts (2022 and beyond) have not yet completed these high-stakes assessments, this study can only capture the performance decline observed in the 2021 cohort, which coincided with stagnation of the case repository, and cannot fully assess the potential compounding effects of emerging AI tools on future standardized test performance. Future research should prioritize multi-center longitudinal designs, incorporate competency-based formative assessments, and establish dynamic teaching quality dashboards to support continuous curriculum optimization [11, 12].

## Conclusions

This 10-year longitudinal study observed that the implementation of clinical case discussions in Systematic Anatomy was associated with improved performance on integrated clinical-anatomy questions in national standardized examinations, and that performance remained stable despite substantial enrollment expansion. The observed decline in the 2021 cohort, which coincided with stagnation of the case repository, suggests that periodic content renewal may be important for sustaining the improved performance observed with case-based instruction. However, given the retrospective single-institution design, these associations cannot be interpreted as causal, and the contribution of unmeasured confounders cannot be excluded.

These findings may inform future curriculum development in foundational sciences. Specifically, further research is needed to evaluate the long-term sustainability of case-based pedagogy, the optimal frequency of case repository updates, and the potential impact of emerging technologies such as generative AI on academic integrity and authentic learning engagement.

## Supplementary Information

Additional file 1.

Additional file 2.

Additional file 3.

Additional file 4.

## Data Availability

All data supporting the findings of this study are available within the paper and its Supplementary Information. Specifically, the anonymized data extracts of national examination performance relevant to the findings of this study are provided in the Supplementary Information files.

## Abbreviations

AI: Artificial intelligence
CBL: Case-based learning
