# Supplementary material for: Impact of clinical case discussions in anatomy on national exam performance: a 10-year longitudinal cohort study
Source: BMC Med Educ. 2026 Aug 27;26:1665. doi: 10.1186/s12909-026-10288-6 (PMC13675961; doi:10.1186/s12909-026-10288-6)
Supplement: Supplementary file 1 [file 12909_2026_10288_MOESM1_ESM.pdf]

桂林医学院

2021 年度临床医学专业（本科）水平测试

成绩分析报告

国家医学考试中心

全国医学教育发展中心

第二部分 本校5年制4年级考生成绩情况

本部分所涉及数据均为报考信息中 5 年制 4 年级的考生群体，在本部分简称“本校考生”。全国的统计指标也是基于总体中 5 年制 4 年级的考生计算得出的。

2.1 整体考试成绩情况

桂林医学院共有 5 年制 4 年级考生 500 人参加水平测试医学基本知识考试，该部分考生平均成绩为 177.17，通过人数为 235 人，通过率为 47.00%；497 人参加临床基本技能考试，该部分考生平均成绩为 78.28，通过人数为 489 人，通过率为 98.39%。

2.2 医学基本知识考试情况

2.2.1 整体情况

表8 医学基本知识考试整体情况

	实考人数	最高分	最低分	平均分	标准差	四分位数			通过人数	通过率（%）
						Q1	Q2	Q3		
本校	500	237.00	100.00	177.17	24.40	162.00	177.00	194.00	235	47.00
全国	39158	285.00	100.00	188.53	34.56	165.00	190.00	214.00	23849	60.90

上图掌握率计算方式为本校考生在各模块上的平均分除以满分，体现了本校考生对此模块的掌握水平。将本校掌握率与全国考生的掌握率相比，可看出本校考生对各模块的掌握是否达到了全国平均水平。

2.2.2.2 基础医学部分

2.2.2.2.1 各学科掌握情况

表10 基础医学部分各学科掌握情况

学科	本校掌握率（%）	全国掌握率（%）	百分等级
病理	55.88	60.49	30.53
解剖	59.05	57.80	56.84
生理	58.60	62.67	29.47
生化	66.07	71.28	25.26
药理	58.43	63.99	28.42
免疫	51.88	59.20	32.63
微生物	51.13	57.23	21.05
病生	54.94	58.14	40.00

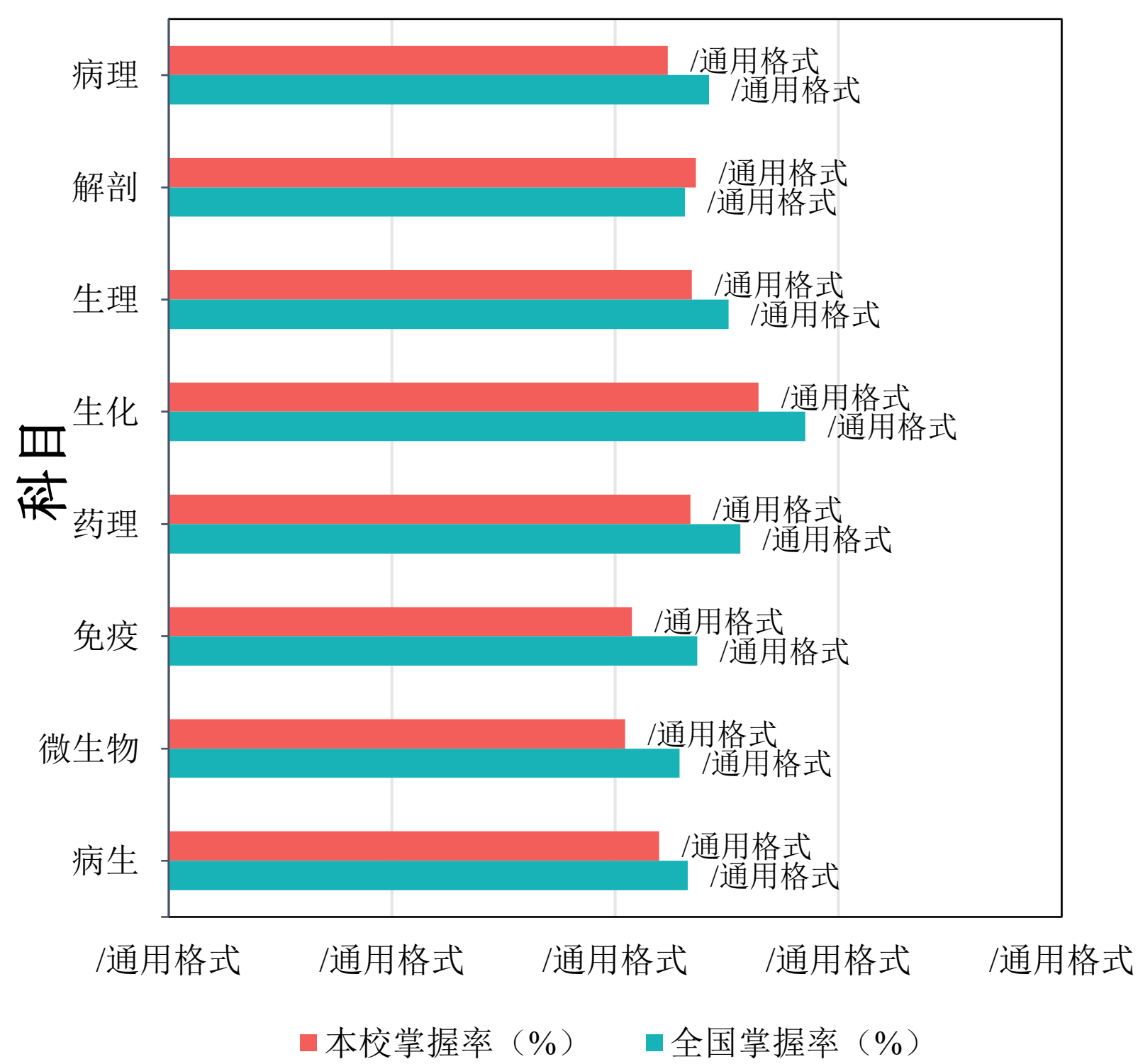

图13 基础医学部分各学科掌握情况

2.2.2.2.4 整合型试题与非整合型试题掌握情况

表13 整合型试题与非整合型试题掌握情况

	整合型试题掌握率（%）		非整合型试题掌握率（%）	
	本校	全国	本校	全国
病理	52.42	57.29	62.40	66.53
解剖	47.24	49.60	64.96	61.90
生理	49.60	54.96	69.40	71.92
生化	75.00	76.67	62.50	69.12
药理	66.52	69.63	51.07	58.86
免疫	44.93	53.67	57.83	63.93
微生物	51.09	55.42	51.18	58.81
病生	48.57	51.93	69.80	72.63

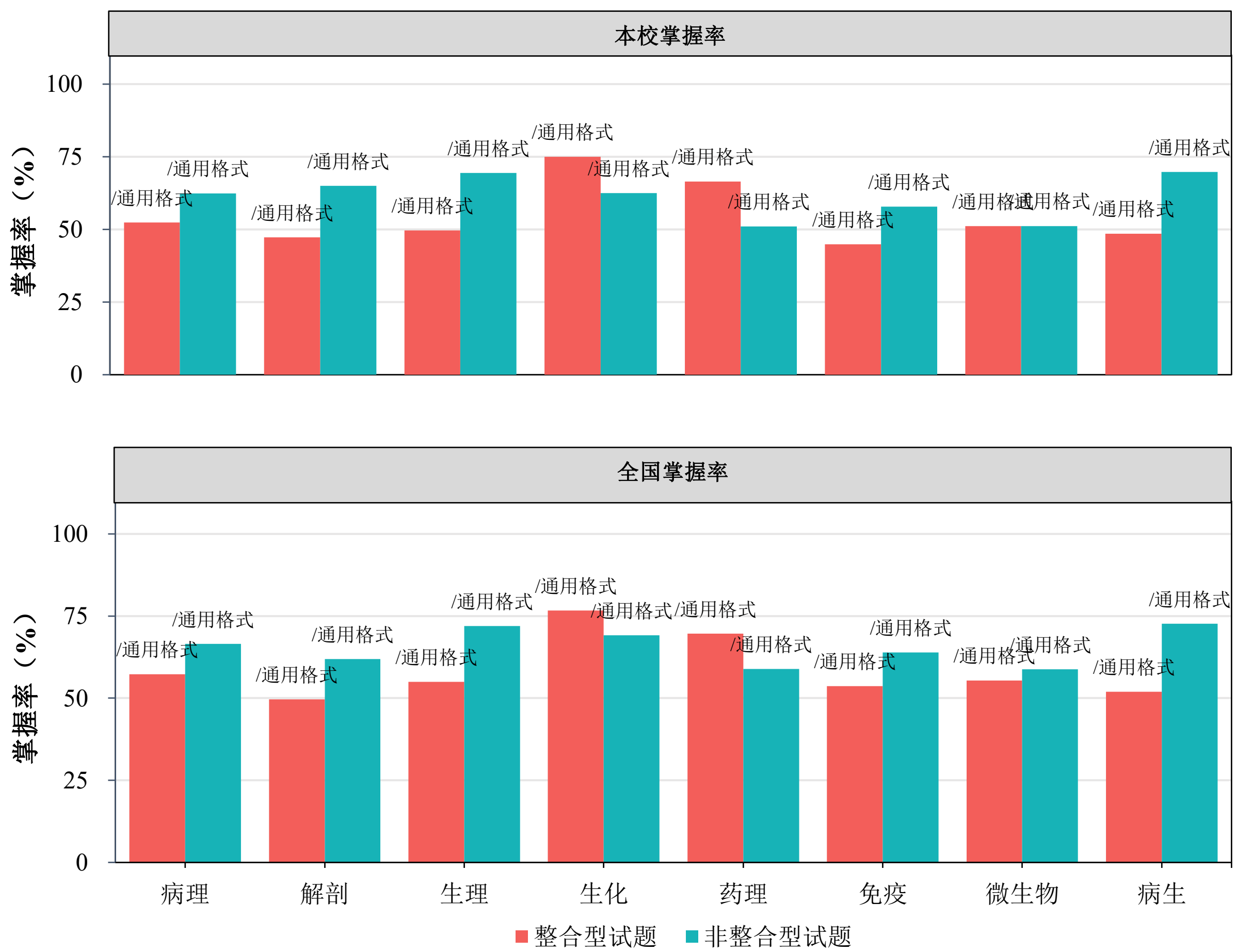

图16 整合型试题与非整合型试题掌握情况

桂林医学院

2022 年度临床医学专业（本科）水平测试

成绩分析报告

国家医学考试中心

全国医学教育发展中心

第二部分 本校 5 年制 4 年级考生成绩情况

本部分所涉及数据均为报考信息中 5 年制 4 年级的考生群体，在本部分简称“本校考生”。全国的统计指标也是基于总体中 5 年制 4 年级的考生计算得出的。

2.1 整体考试成绩情况

桂林医学院共有 5 年制 4 年级考生 754 人参加水平测试医学基本知识考试，该部分考生平均成绩为 186.30，通过人数为 460 人，通过率为 61.01%；749 人参加临床基本技能考试，该部分考生平均成绩为 82.37，通过人数为 747 人，通过率为 99.73%。

2.2 医学基本知识考试情况

2.2.1 整体情况

表8 医学基本知识考试整体情况

	实考人数	最高分	最低分	平均分	标准差	四分位数			通过人数	通过率（%）	百分等级
						Q1	Q2	Q3			
本校	754	259.00	101.00	186.30	26.37	168.00	187.00	204.00	460	61.01	38.60
全国	21826	286.00	3.00	191.85	32.44	170.00	194.00	215.00	14439	66.16	/

上图掌握率计算方式为本校考生在各模块上的平均分除以满分，体现了本校考生对此模块的掌握水平。将本校掌握率与全国考生的掌握率相比，可看出本校考生对各模块的掌握是否达到了全国平均水平。

2.2.2.2 基础医学部分

2.2.2.2.1 各学科掌握情况

表10 基础医学部分各学科掌握情况

学科	本校掌握率（%）	全国掌握率（%）	百分等级
病理	55.20	57.02	49.12
解剖	61.66	60.74	56.14
生理	62.75	66.94	26.32
生化	61.22	65.28	22.81
药理	55.10	57.40	35.09
免疫	41.63	47.12	24.56
微生物	63.33	68.60	26.32
病生	61.60	61.30	61.40

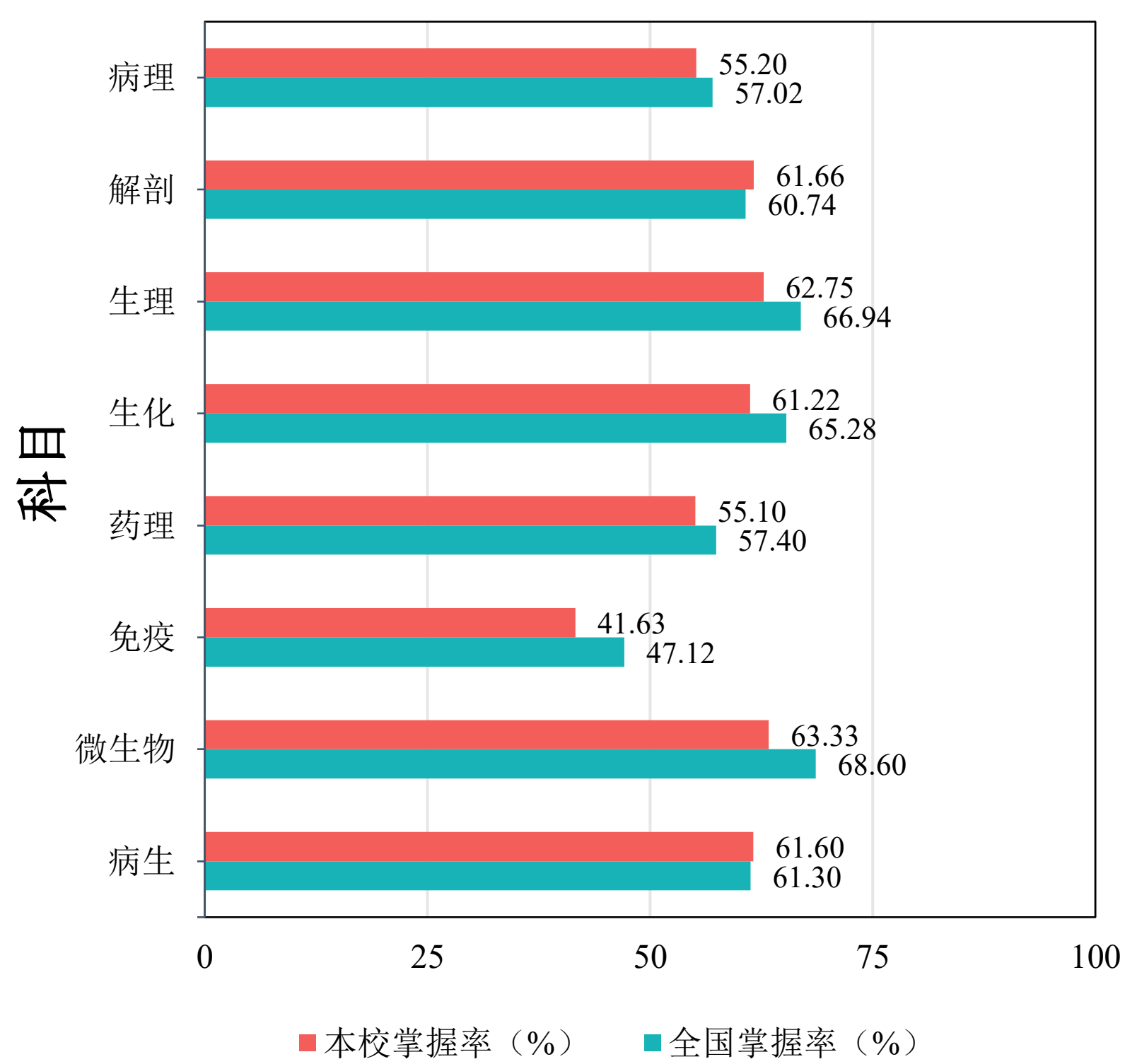

图13 基础医学部分各学科掌握情况

2.2.2.2.4 整合型试题与非整合型试题掌握情况

表13 整合型试题与非整合型试题掌握情况

	整合型试题			非整合型试题		
	本校掌握率（%）	全国掌握率（%）	百分等级	本校掌握率（%）	全国掌握率（%）	百分等级
病理	57.26	58.86	49.12	50.62	52.93	42.11
解剖	68.10	64.73	68.42	57.37	58.08	45.61
生理	65.19	69.86	31.58	59.08	62.57	24.56
生化	78.68	80.78	31.58	54.23	59.08	19.30
药理	57.64	59.94	35.09	52.56	54.86	45.61
免疫	47.94	50.37	35.09	36.89	44.68	21.05
微生物	62.64	65.32	38.60	63.84	71.07	10.53
病生	61.72	62.74	45.61	61.44	59.15	73.68

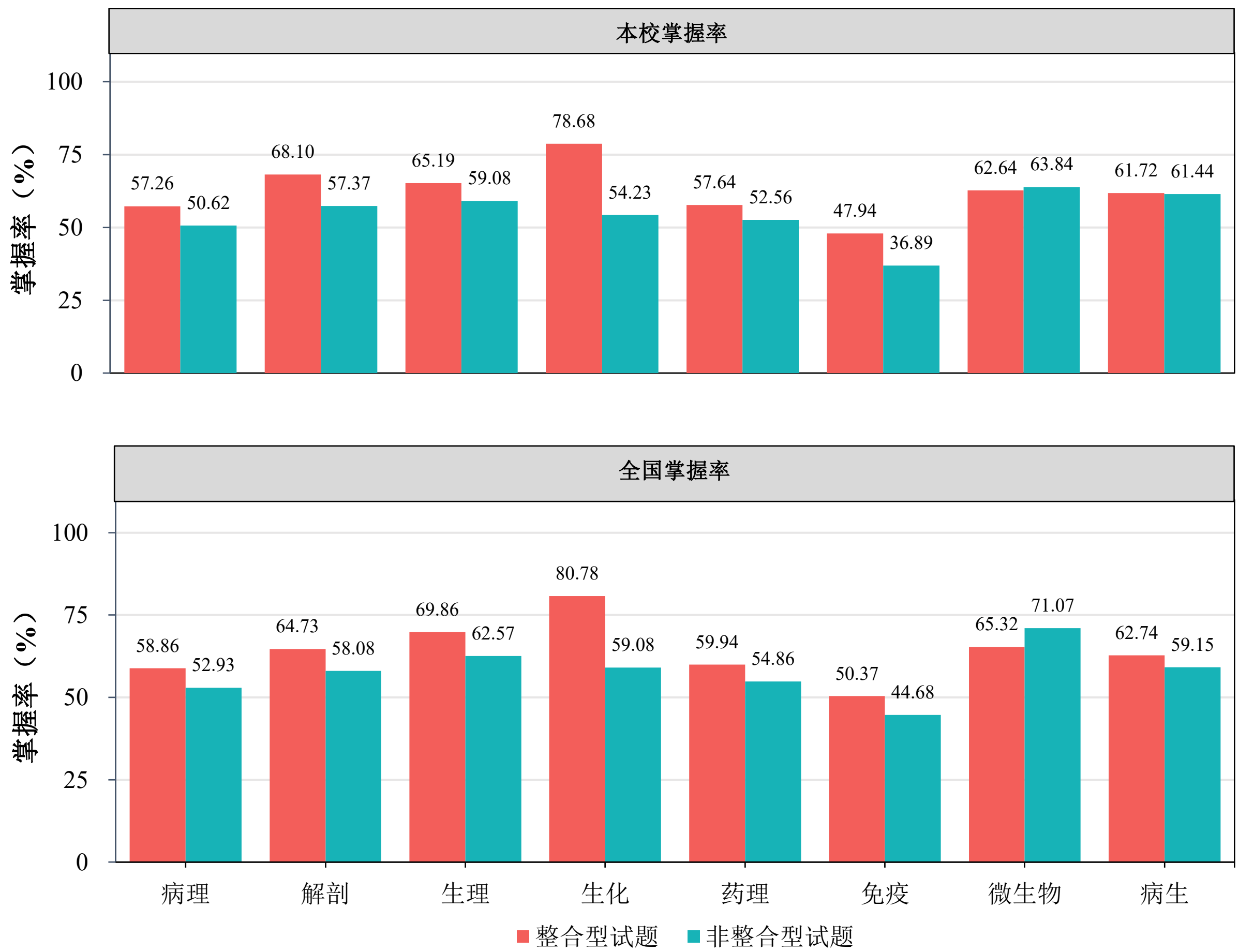

图16 整合型试题与非整合型试题掌握情况

桂林医学院

2023 年度临床医学专业（本科）水平测试

成绩分析

国家医学考试中心

全国医学教育发展中心

第二部分 本校 5 年制 4 年级考生成绩情况

本部分所涉及数据均为报考信息中 5 年制 4 年级的考生群体，在本部分简称“本校考生”。全国的统计指标也是基于总体中 5 年制 4 年级的考生计算得出的。

2.1 整体考试成绩情况

桂林医学院共有 5 年制 4 年级考生 795 人参加水平测试医学基本知识考试，该部分考生平均成绩为 188.92，通过人数为 508 人，通过率为 63.90%；780 人参加临床基本技能考试，该部分考生平均成绩为 81.41，通过人数为 686 人，通过率为 87.95%。

2.2 医学基本知识考试情况

2.2.1 整体情况

表8 医学基本知识考试整体情况

	实考人数	最高分	最低分	平均分	标准差	四分位数			通过人数	通过率（%）	百分等级
						Q1	Q2	Q3			
本校	795	267.00	107.00	188.92	26.92	170.00	189.00	208.00	508	63.90	47.87
全国	41115	286.00	24.00	191.07	33.08	168.00	192.00	215.00	26311	63.99	/

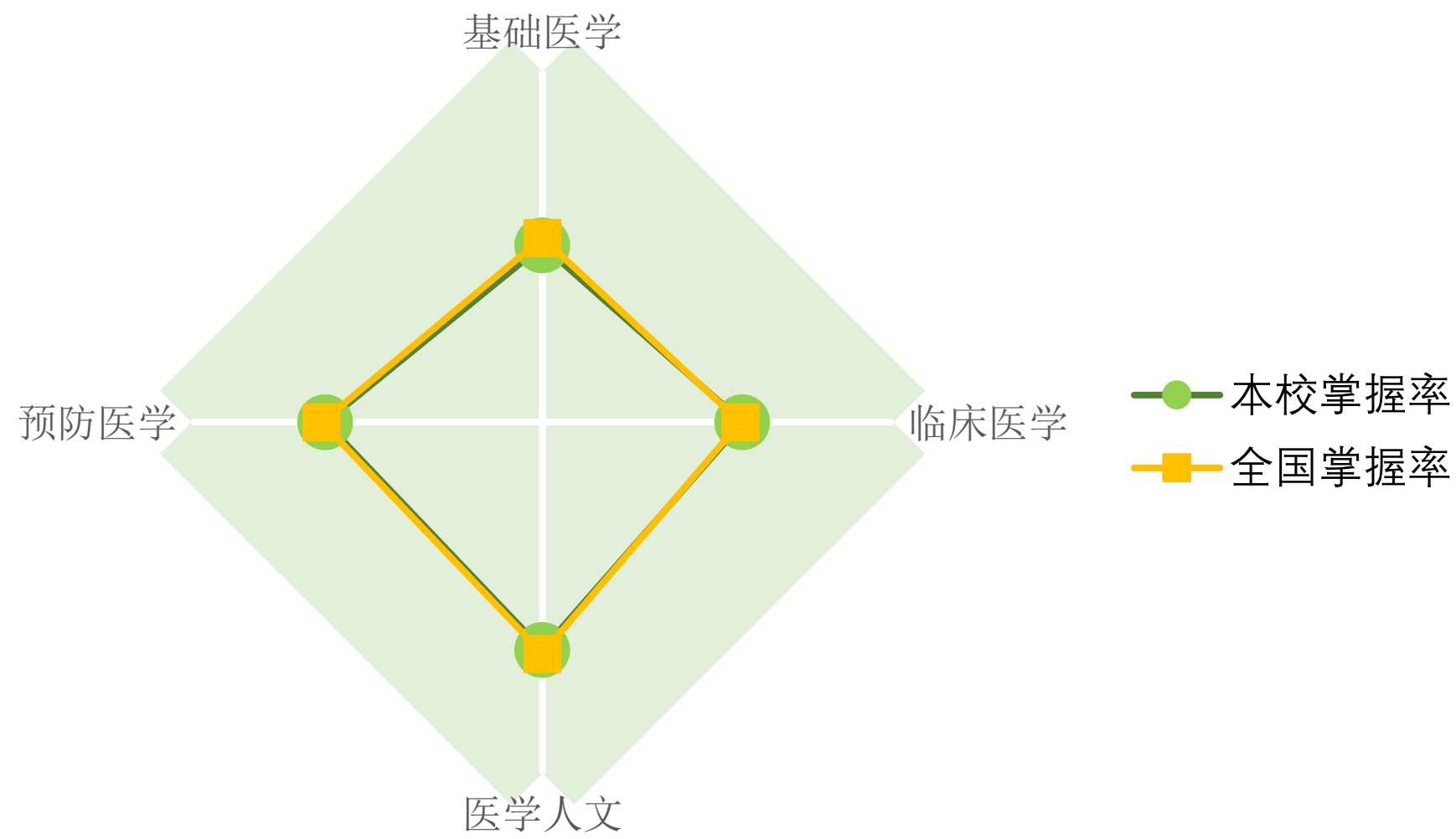

图12 本校考生不同模块掌握情况

上图掌握率计算方式为本校考生在各模块上的平均分除以满分，体现了本校考生对此模块的掌握水平。将本校掌握率与全国考生的掌握率相比，可看出本校考生对各模块的掌握是否达到了全国平均水平。

2.2.2.2 基础医学部分

2.2.2.2.1 各学科掌握情况

表10 基础医学部分各学科掌握情况

学科	本校掌握率（%）	全国掌握率（%）	百分等级
病理	45.47	48.28	40.43
解剖	51.11	48.51	64.89
生理	48.93	52.75	26.60
生化	54.39	55.98	36.17
药理	44.60	48.10	30.85
免疫	50.58	54.18	31.91
微生物	59.75	61.85	36.17
病生	59.92	60.90	42.55

表13 整合型试题与非整合型试题掌握情况

	整合型试题			非整合型试题		
	本校掌握率（%）	全国掌握率（%）	百分等级	本校掌握率（%）	全国掌握率（%）	百分等级
病理	46.43	47.09	52.13	43.34	50.92	20.21
解剖	42.35	40.22	62.77	56.95	54.03	68.09
生理	37.13	43.40	22.34	60.73	62.09	37.23
生化	64.94	65.31	48.94	50.18	52.25	34.04
药理	53.72	54.17	47.87	37.00	43.04	17.02
免疫	39.79	44.34	27.66	58.66	61.55	38.30
微生物	67.80	68.10	45.74	53.71	57.17	25.53
病生	62.39	62.76	44.68	56.23	58.12	41.49

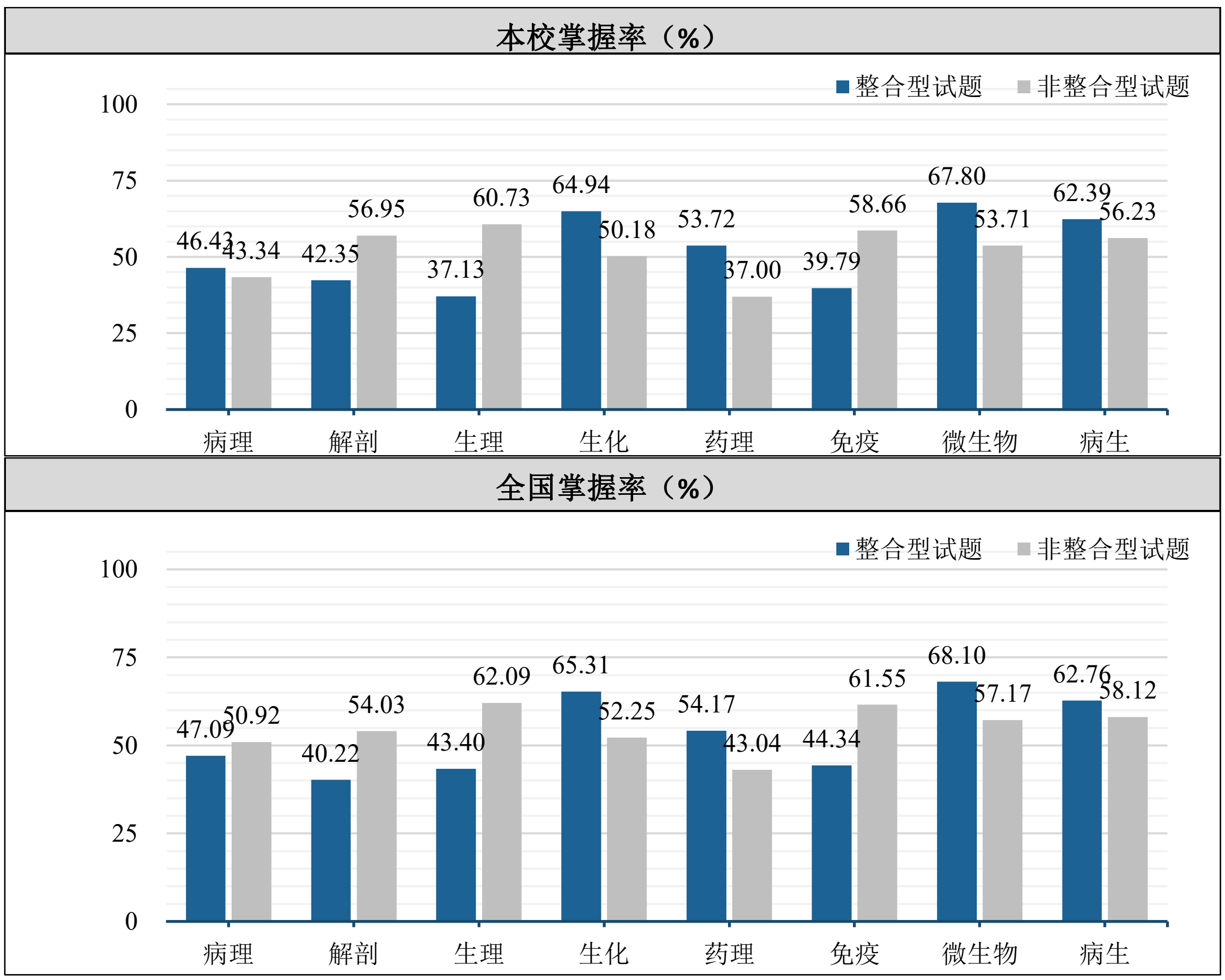

图16 整合型试题与非整合型试题掌握情况

2024年度临床医学专业（本科）水平测试成绩分析报告

桂林医学院

全国医学教育发展中心　国家医学考试中心

第二部分 本校考生成绩情况

本部分所涉及数据均为报考信息中即将进入临床实习年级的临床医学类专业本科生，在本部分简称“本校考生”。全国的统计指标也是基于此范围计算得出的。

一、 整体考试成绩情况

桂林医学院的医学基本知识及临床基本技能两门考试的总通过率情况如下表所示：

表8 本校整体考试情况

专业	学制	实考人数[3]	总通过率（%）
临床医学	5 年制	746	48.92
医学影像学	5 年制	80	40.00
儿科学	5 年制	57	43.86
**本校整体**		**883**	**47.79**

桂林医学院共有 900 人参加水平测试医学基本知识考试，该部分考生平均成绩为 181.37，通过人数为 475 人，通过率为 52.78%；881 人参加临床基本技能考试，该部分考生平均成绩为 79.08，通过人数为 684 人，通过率为 77.64%。本校各学制考试情况如下表所示：

表9 本校医学基本知识考试分专业学制考试情况

专业	学制	实考人数	平均分	最高分	最低分	通过人数	通过率（%）
临床医学	5 年制	763	181.90	258.00	82.00	407	53.34
医学影像学	5 年制	80	180.10	239.00	111.00	41	51.25
儿科学	5 年制	57	176.14	228.00	120.00	27	47.37
**本校整体**		**900**	**181.37**	**258.00**	**82.00**	**475**	**52.78**

[3] 此表“实考人数”是指同时参与医学基本知识与临床基本技能两门考试的考生群体。

2. 各专业与学制考生不同模块掌握情况

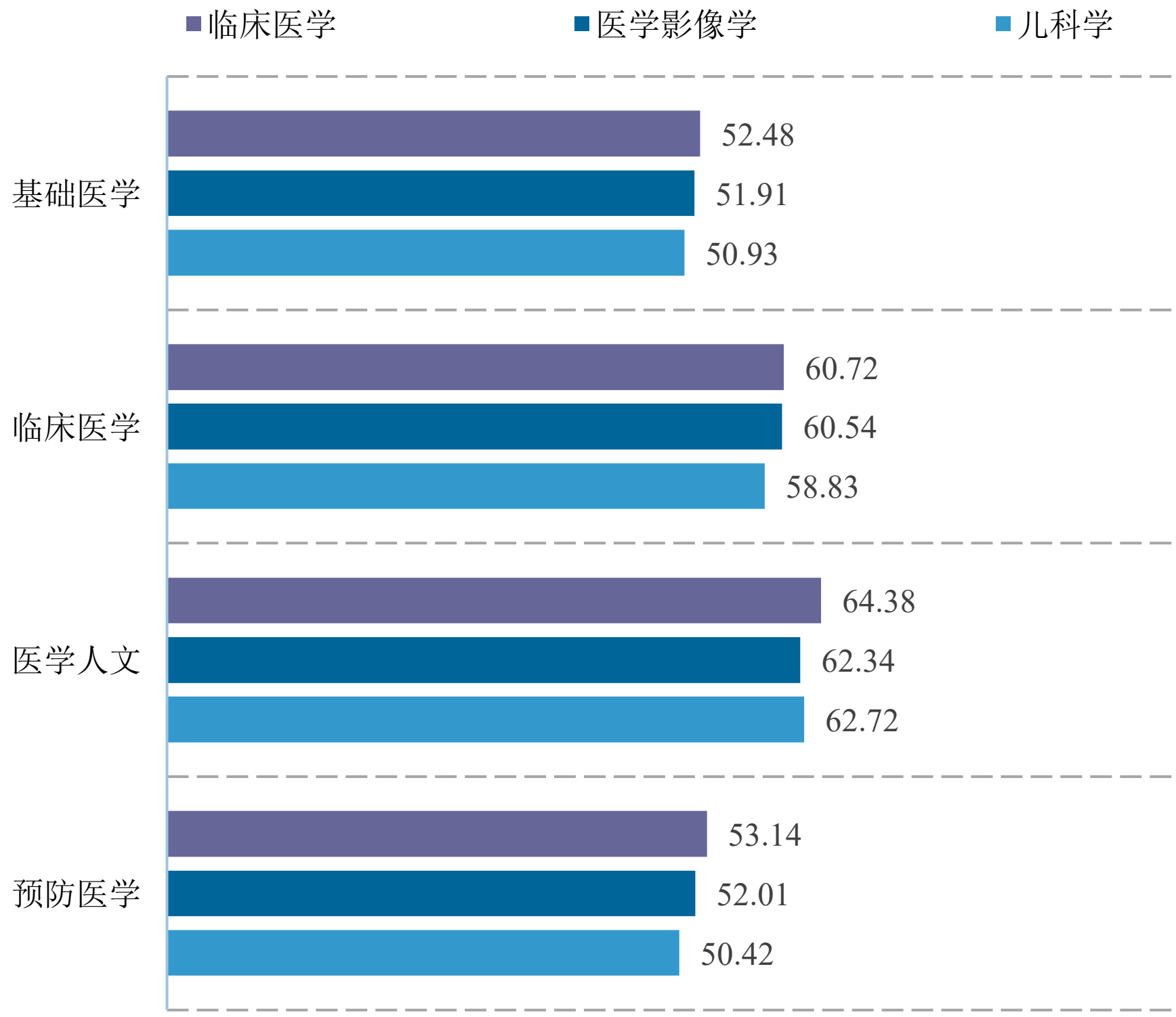

图9 各专业考生不同模块掌握情况（%）

表13 各专业与学制考生不同模块掌握情况（%）

专业	学制	基础医学	临床医学	医学人文	预防医学
临床医学	5 年制	52.48	60.72	64.38	53.14
医学影像学	5 年制	51.91	60.54	62.34	52.01
儿科学	5 年制	50.93	58.83	62.72	50.42

(三) 基础医学部分

1. 各学科掌握情况

（1）总体情况

表14 基础医学部分各学科掌握情况

学科	本校掌握率（%）	全国掌握率（%）	百分等级
病理	57.75	58.83	50.46
解剖	58.52	55.14	72.48
生理	57.65	61.11	25.69
生化	65.79	67.53	39.45

表15 各专业与学制考生不同学科掌握情况（%）

专业	学制	病理	解剖	生理	生化	药理	免疫	微生物	病生
临床医学	5 年制	57.33	58.88	57.50	65.91	41.60	43.21	44.48	51.46
医学影像学	5 年制	60.53	56.92	58.65	65.98	41.00	38.04	44.29	46.70
儿科学	5 年制	59.51	55.91	58.19	63.91	40.00	38.97	40.10	48.33

2. 不同认知层次掌握情况

（1）总体情况

表16 基础医学部分不同认知层次掌握情况

认知层次	本校掌握率（%）	全国掌握率（%）	百分等级
记忆	50.93	52.50	44.95
理解	56.02	58.15	31.19
简单应用	49.04	50.57	44.04
综合应用	66.91	66.60	54.13

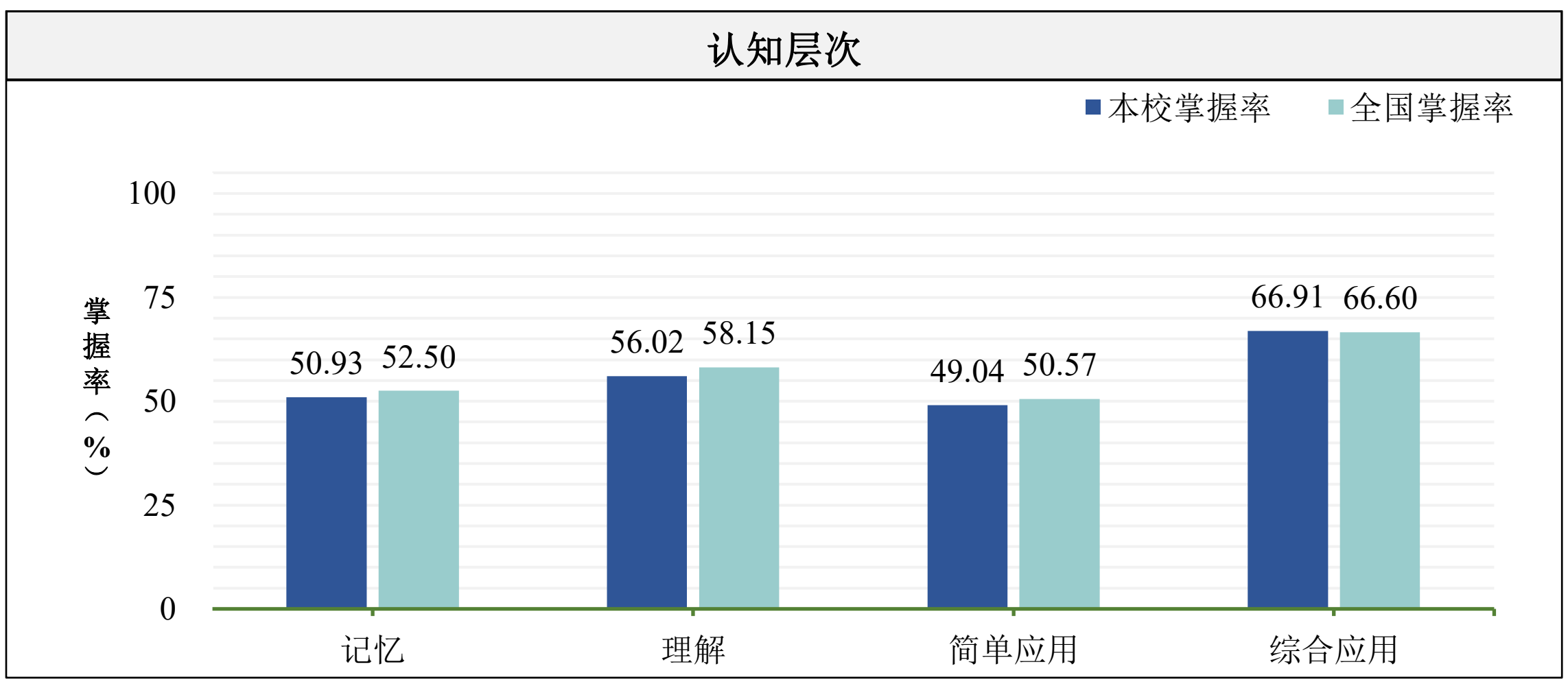

图12 基础医学部分不同认知层次掌握情况

	整合型试题			非整合型试题		
	本校掌握率（%）	全国掌握率（%）	百分等级	本校掌握率（%）	全国掌握率（%）	百分等级
解剖	56.02	54.70	58.72	60.71	55.53	78.90
生理	43.85	47.44	30.28	71.44	74.77	26.61
生化	73.08	70.66	70.64	62.88	66.28	26.61
药理	36.58	39.15	34.86	48.74	52.50	21.10
免疫	42.54	44.82	37.61	42.44	44.93	37.61
微生物	44.01	46.95	33.94	44.41	47.43	38.53
病生	50.91	51.00	55.05	50.76	56.04	30.28

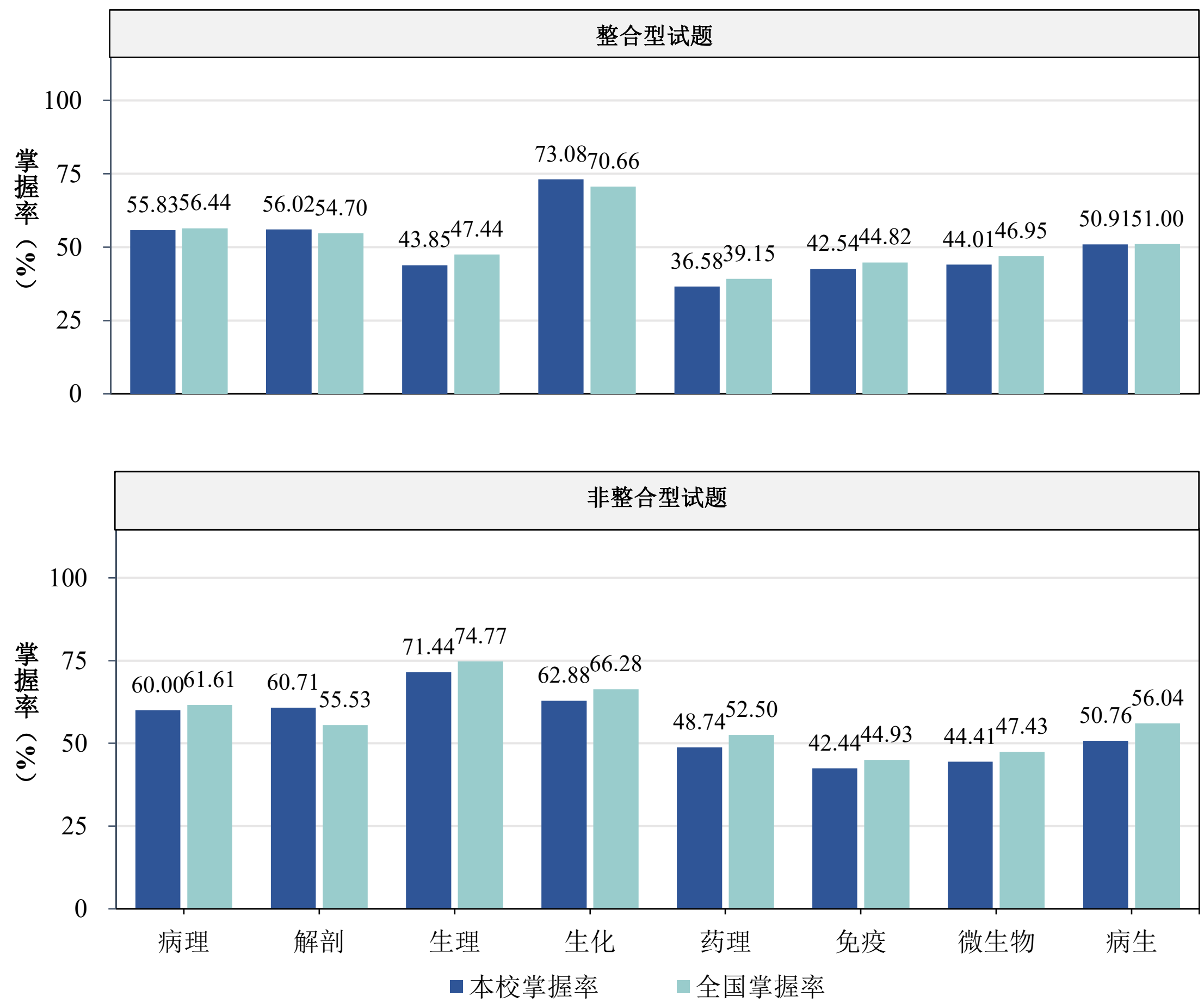

图16 整合型试题与非整合型试题掌握情况

（2）各专业与学制考生不同类别题型掌握情况

表21 各专业与学制考生整合型试题与非整合型试题掌握情况（1）（%）

专业	学制	病理		解剖		生理		生化	
		整合	非整合	整合	非整合	整合	非整合	整合	非整合
临床医学	5年制	55.56	59.39	55.96	61.44	43.91	71.10	73.07	63.05
医学影像学	5年制	56.96	64.69	56.61	57.19	43.75	73.54	73.44	63.00
儿科学	5年制	57.77	61.55	55.89	55.92	43.27	73.10	72.81	60.35

表22 各专业与学制考生整合型试题与非整合型试题掌握情况（2）（%）

专业	学制	药理		免疫		微生物		病生	
		整合	非整合	整合	非整合	整合	非整合	整合	非整合
临床医学	5年制	36.75	48.87	43.21	43.22	44.25	44.78	51.77	51.09
医学影像学	5年制	35.83	48.75	36.25	39.38	43.91	44.79	44.17	49.75
儿科学	5年制	35.38	46.93	42.40	36.40	41.01	38.89	48.83	47.72

(四) 临床医学部分

1. 各学科掌握情况

（1）总体情况

表23 临床医学部分各学科掌握情况

学科	本校掌握率（%）	全国掌握率（%）	百分等级
内科学	62.38	63.65	44.95
外科学	64.69	64.98	47.71
妇产科学	48.78	49.66	54.13
儿科学	53.73	55.35	45.87

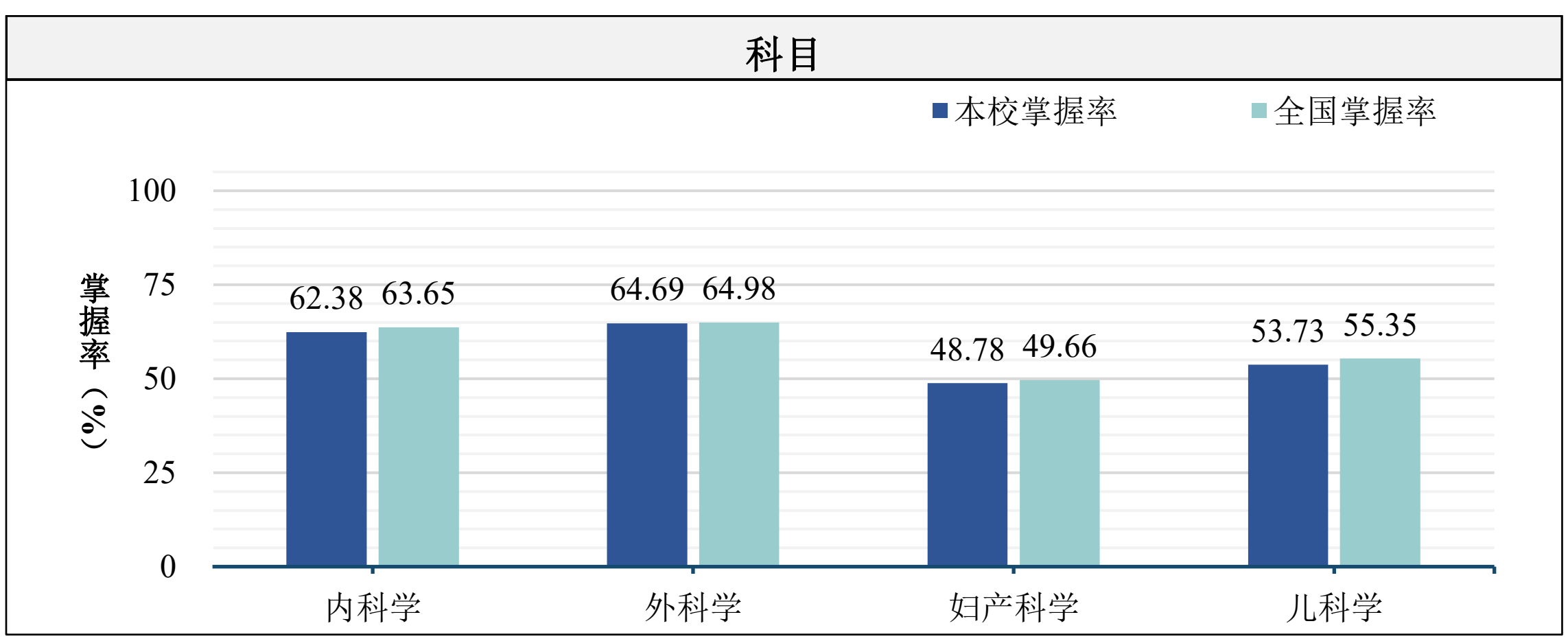

图17 临床医学部分各学科掌握情况

桂林医科大学

2025年临床医学专业(本科)水平测试

成绩分析报告

全国医学教育发展中心　　国家医学考试中心

第二部分 本校考生成绩情况

本部分所涉及数据均以报考信息中**完成基础和临床阶段理论学习和见习，拟进入临床实习的临床医学类专业学生**为样本。全国的统计指标也是基于此范围计算得出的。

一、 整体考试成绩情况

学校医学基本知识及临床基本技能两门考试的总通过率情况如下表所示：

表 8 本校整体考试情况

专业	学制	实考人数[3]	总通过率（%）
临床医学	5 年制	708	61.58
医学影像学	5 年制	89	30.34
儿科学	5 年制	78	42.31
**本校整体**		**875**	**56.69**

医学基本知识考试实考人数为 875 人，该部分考生平均成绩为 187.75，通过人数为 557 人，通过率为 63.66%；临床基本技能考试实考人数为 875 人，该部分考生平均成绩为 81.05，通过人数为 729 人，通过率为 83.31%。本校各专业学制考试情况如下表所示：

表 9 本校医学基本知识考试分专业学制考试情况

专业	学制	实考人数	平均分	最高分	最低分	通过人数	通过率（%）	百分等级
临床医学	5 年制	708	191.41	255.00	94.00	484	68.36	55.37
医学影像学	5 年制	89	168.16	240.00	105.00	34	38.20	17.14
儿科学	5 年制	78	176.96	229.00	134.00	39	50.00	13.64
**本校整体**		**875**	**187.75**	**255.00**	**94.00**	**557**	**63.66**	**46.83**

[3] 此表"实考人数"是指同时参与医学基本知识与临床基本技能两门考试的考生群体。

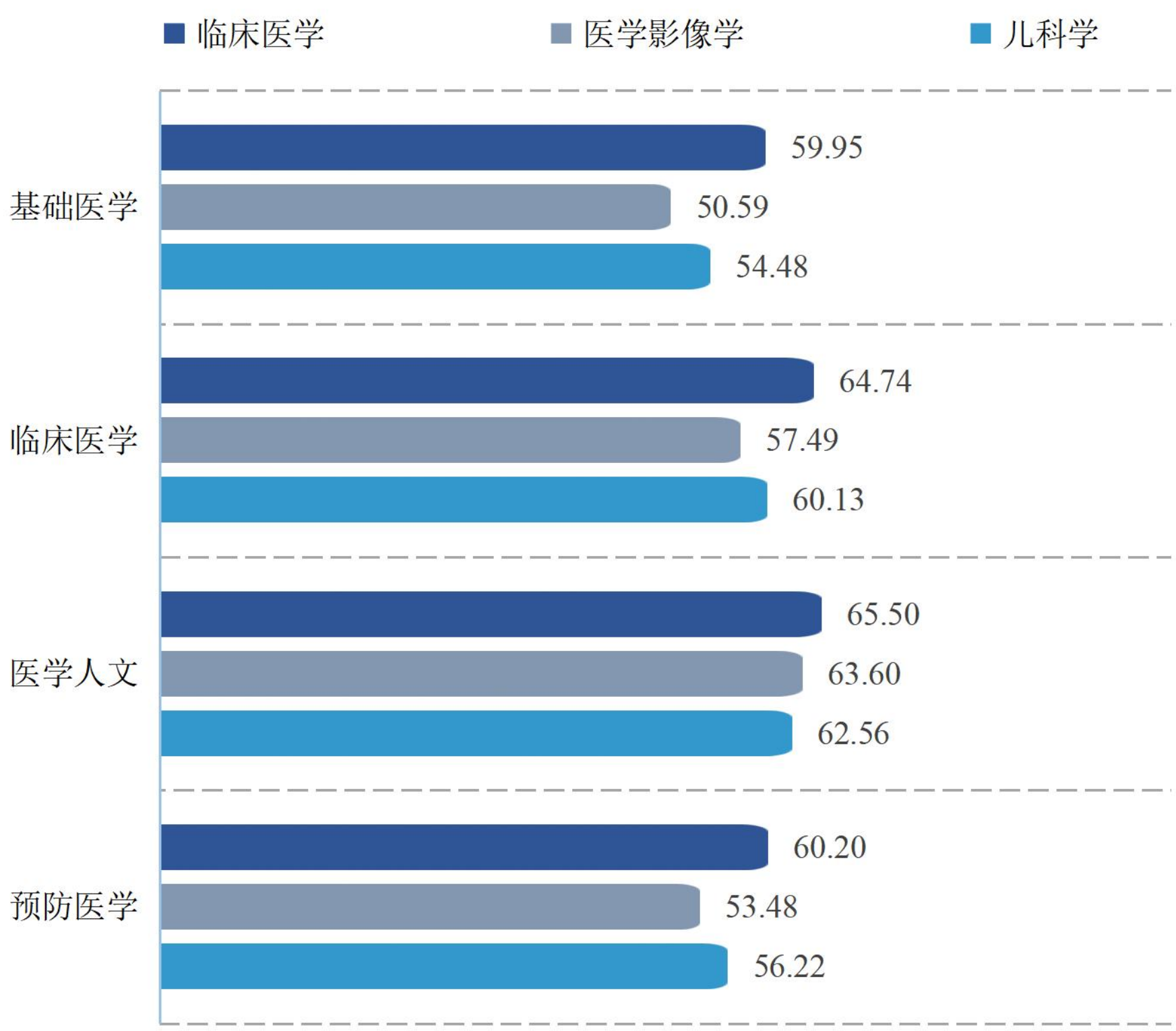

图 9 各专业考生不同模块掌握情况（%）

表 13 各专业与学制考生不同模块掌握情况（%）

专业	学制	基础医学	临床医学	医学人文	预防医学
临床医学	5 年制	59.95	64.74	65.50	60.20
医学影像学	5 年制	50.59	57.49	63.60	53.48
儿科学	5 年制	54.48	60.13	62.56	56.22

1. 基础医学部分

（1）各学科掌握情况

a）总体情况

表 14 基础医学部分各学科掌握情况

学科	本校掌握率（%）	全国掌握率（%）	百分等级
病理	61.03	61.49	50.79
解剖	49.11	51.86	38.89
生理	66.50	65.53	61.11
生化	61.48	62.68	47.62
药理	65.16	66.09	48.41

表15 各专业与学制考生不同学科掌握情况（%）

专业	学制	病理	解剖	生理	生化	药理	免疫	微生物	病生
临床医学	5年制	62.65	49.72	68.41	62.69	67.19	50.56	53.73	61.83
医学影像学	5年制	51.87	46.55	56.55	52.01	55.37	39.97	48.57	52.71
儿科学	5年制	56.78	46.52	60.58	61.26	57.83	44.60	51.08	55.36

（2）不同认知层次掌握情况

a）总体情况

表16 基础医学部分不同认知层次掌握情况

认知层次	本校掌握率（%）	全国掌握率（%）	百分等级
记忆	56.95	58.41	47.62
理解	60.56	61.44	48.41
简单应用	57.11	58.90	42.86
综合应用	59.95	60.33	53.97

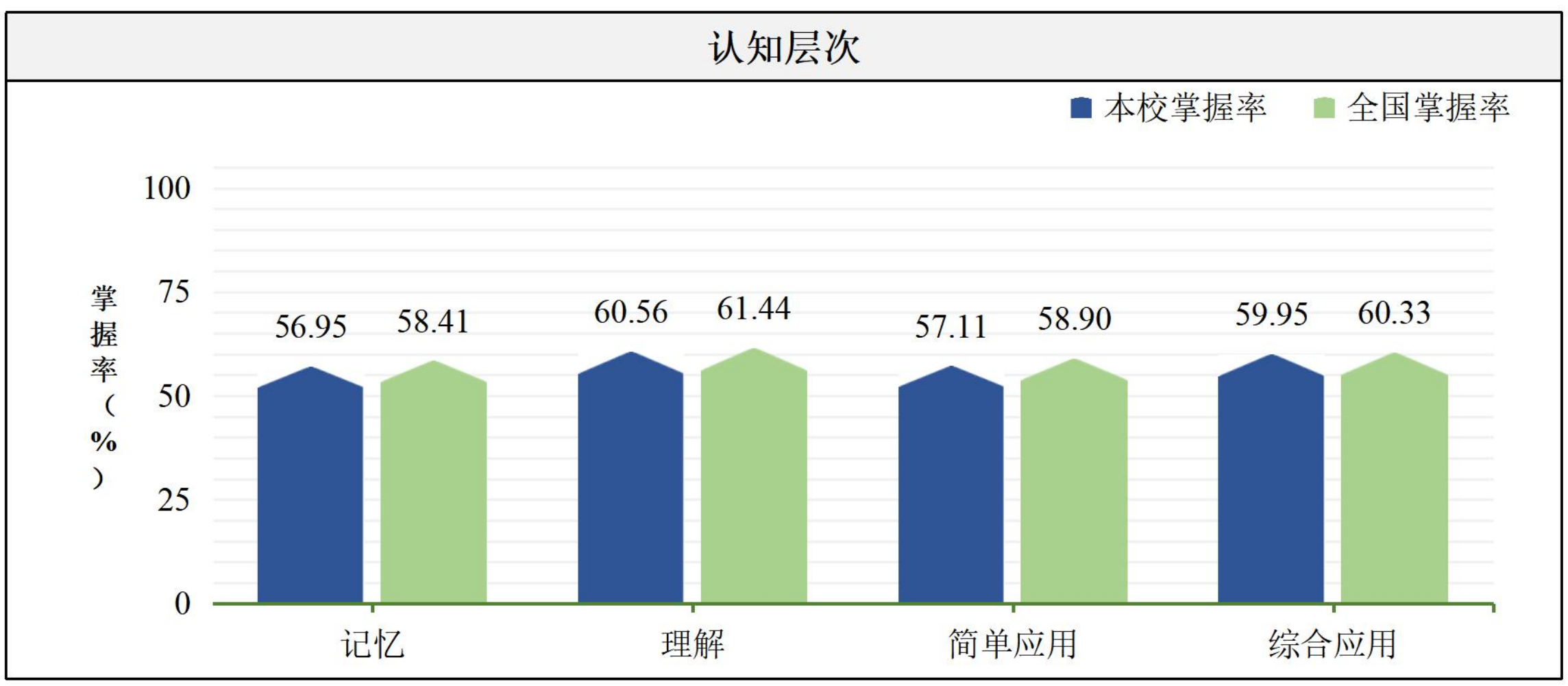

图12 基础医学部分不同认知层次掌握情况

学科	整合型试题			非整合型试题		
	本校掌握率（%）	全国掌握率（%）	百分等级	本校掌握率（%）	全国掌握率（%）	百分等级
病理	57.58	57.87	53.17	65.11	65.77	46.83
解剖	49.16	55.22	23.02	49.06	48.49	61.11
生理	53.49	53.71	53.97	79.52	77.35	64.29
生化	72.34	70.53	72.22	57.13	59.54	38.89
药理	70.15	71.83	42.06	58.91	58.91	54.76
免疫	36.54	43.88	20.63	61.37	65.67	34.92
微生物	50.47	52.16	47.62	55.89	56.92	47.62
病生	54.90	52.12	73.81	66.83	65.64	58.73

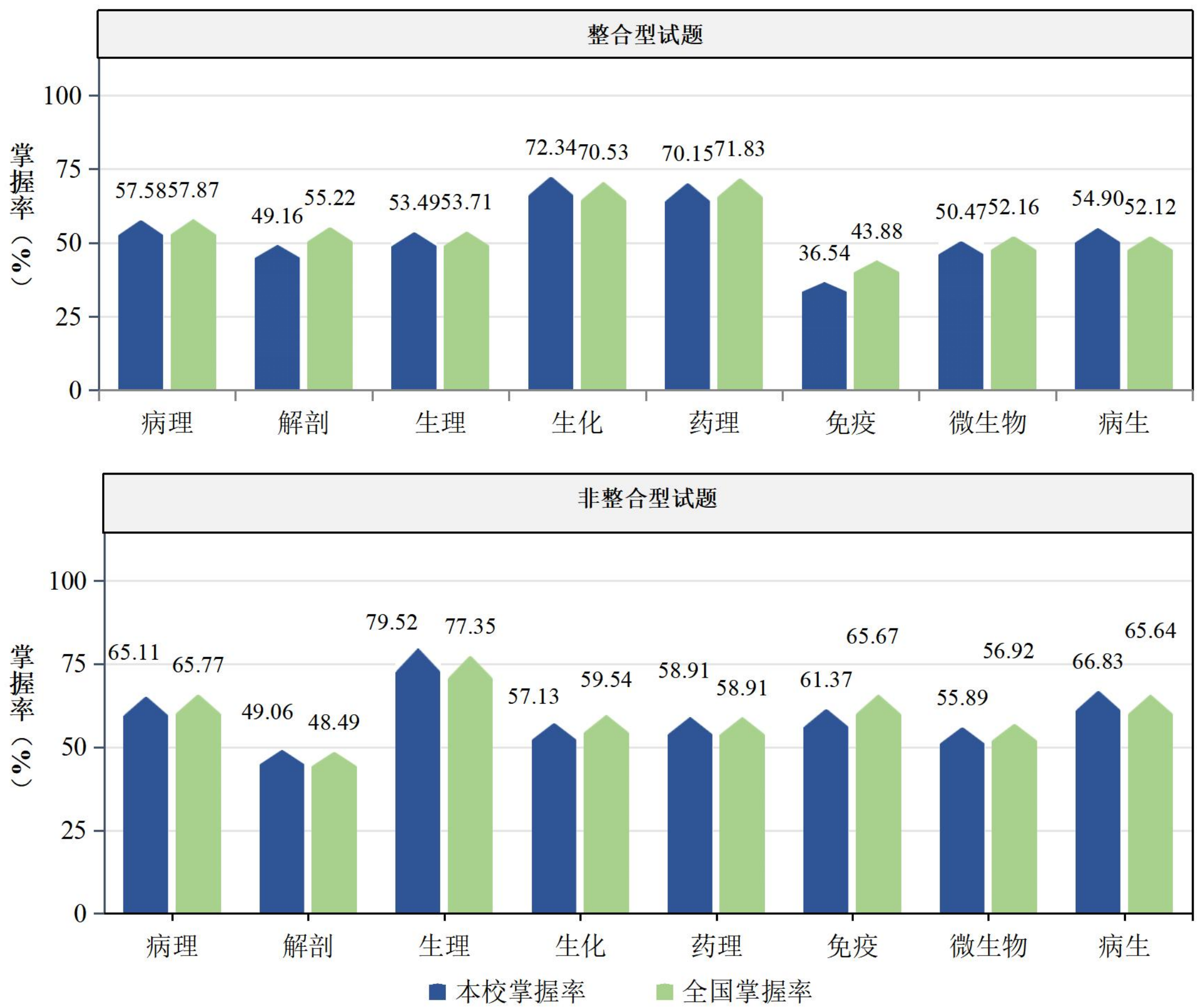

图16 整合型试题与非整合型试题掌握情况

b）各专业与学制考生不同类别题型掌握情况

表21 各专业与学制考生整合型试题与非整合型试题掌握情况（1）（%）

专业	学制	病理	解剖	生理	生化

		整合	非整合	整合	非整合	整合	非整合	整合	非整合
临床医学	5年制	59.35	66.55	49.88	49.56	55.39	81.43	73.31	58.45
医学影像学	5年制	49.27	54.95	44.46	48.64	43.45	69.66	61.24	48.31
儿科学	5年制	50.99	63.64	47.99	45.05	47.65	73.50	76.28	55.26

表22 各专业与学制考生整合型试题与非整合型试题掌握情况（2）（%）

专业	学制	药理		免疫		微生物		病生	
		整合	非整合	整合	非整合	整合	非整合	整合	非整合
临床医学	5年制	72.13	61.02	37.45	63.68	51.45	56.38	55.84	69.01
医学影像学	5年制	59.55	50.14	30.66	49.28	45.91	51.69	48.88	57.30
儿科学	5年制	64.23	49.84	34.98	54.21	46.70	56.20	53.21	57.95

2. 临床医学部分

（1）各学科掌握情况

a）总体情况

表23 临床医学部分各学科掌握情况

学科	本校掌握率（%）	全国掌握率（%）	百分等级
内科学	68.32	67.37	61.11
外科学	62.55	64.05	46.83
妇产科学	48.10	50.79	46.03
儿科学	60.01	61.80	45.24

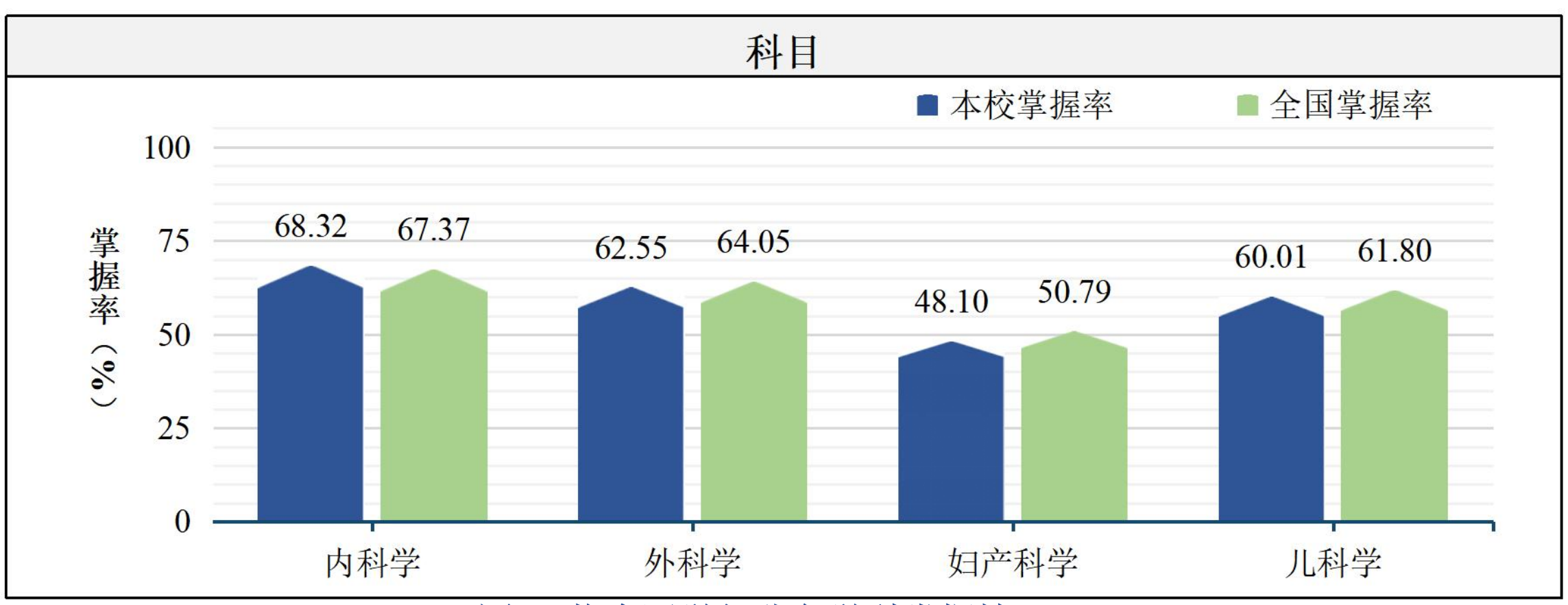

图17 临床医学部分各学科掌握情况
