# Supplementary material for: Impact of clinical case discussions in anatomy on national exam performance: a 10-year longitudinal cohort study
Source: BMC Med Educ. 2026 Aug 27;26:1665. doi: 10.1186/s12909-026-10288-6 (PMC13675961; doi:10.1186/s12909-026-10288-6)
Supplement: Supplementary file 2 [file 12909_2026_10288_MOESM2_ESM.pdf]

2021年医师资格考试
成绩分析报告

（桂林医学院，临床执业医师）

国家医学考试中心

前 言

医师资格考试是根据《中华人民共和国执业医师法》设置的执业资格考试。作为卫生行业的重要准入标准，医师资格考试不仅关系卫生行业从业人员的准入与管理，而且对医学教育改革与发展有重要的影响。

随着医师资格考试制度的不断完善和医学教育改革的不断深入，为满足医学教育发展与改革的需要，密切医师资格考试与医学教育的互动关系，向医学教育提供相关服务，我们编制了《2021年医师资格考试成绩分析报告（临床执业医师）》（以下简称《报告》）。

《报告》分三部分。第一部分为桂林医学院（以下简称本校）全体考生参加2021年临床执业医师考试的基本情况，包括考生构成情况，考试通过情况和考生成绩描述，其中全体考生是指实践技能考试实考考生，包括应届考生和非应届考生，并包含不同学历层次的考生；应届本科生指毕业1年的本科考生，应届研究生指毕业当年的硕士或博士研究生考生。第二部分为本校全日制应届本科考生参加2021年临床执业医师考试的整体情况与全国这一群体考试情况的比较。第三部分为本校全日制应届本科考生参加2021年临床执业医师考试医学综合考试的基本情况，包括各模块、学科、各认知层次的得分掌握情况等。

《报告》的数据来源以考生个人填报的报名信息为基础，包含一试和延考考生的成绩，不包含二次考试的考生成绩。医学综合考试通过人数以四个单元成绩统计，不含加试（军事医学、院前急救、儿科）的成绩。

第二部分

本校全日制应届本科考生

考试情况比较

报告对象：桂林医学院2020年毕业并参加2021年医师资格考试的全日制本科考生；全国2020年毕业并参加2021年医师资格考试的全日制本科考生。

考生群体	实践实考人数	实践通过人数	实践通过率（%）	综合考试实考人数	综合考试通过人数	综合考试通过率（%）	总通过率（%）
本校	438	411	93.84	408	305	74.75	69.63
全国	70733	62279	88.05	61299	46312	75.55	65.47

*注：综合考试即医学综合考试。

3.3 学科掌握情况

3.3.1 基础医学

表3-3 基础医学各学科掌握情况

学科	考生群体	平均分	标准差	最高分	最低分	掌握率（%）
病理生理学	本校	4.63	1.45	9	1	51.44
	辖区内学校	4.68	1.43	9	1	52.00
	全国	4.87	1.58	9	0	54.11
病理学	本校	10.58	2.22	15	5	70.53
	辖区内学校	10.69	2.22	15	3	71.27
	全国	10.47	2.43	15	0	69.80
解剖学	本校	3.96	1.33	7	0	44.00
	辖区内学校	3.84	1.29	8	0	42.67
	全国	3.69	1.35	9	0	41.00
生理学	本校	8.40	2.19	14	2	60.00
	辖区内学校	8.45	2.14	14	2	60.36
	全国	8.91	2.19	14	0	63.64
生物化学	本校	6.38	1.80	11	1	58.00
	辖区内学校	6.40	1.70	11	1	58.18
	全国	6.52	1.90	11	0	59.27
药理学	本校	4.69	1.48	8	0	58.63
	辖区内学校	4.77	1.48	8	0	59.63
	全国	4.57	1.53	8	0	57.13
医学免疫学	本校	4.66	1.25	7	0	66.57
	辖区内学校	4.86	1.23	7	0	69.43
	全国	4.72	1.36	7	0	67.43
医学微生物学	本校	4.73	1.18	7	2	67.57
	辖区内学校	4.66	1.24	7	0	66.57
	全国	4.55	1.30	7	0	65.00

2022 年医师资格考试
成绩分析报告

（桂林医学院，临床执业医师）

国家医学考试中心

第二部分

本校全日制应届本科考生

考试情况比较

报告对象：桂林医学院 2021 年毕业并参加 2022 年医师资格考试的全日制本科考生；

全国 2021 年毕业并参加 2022 年医师资格考试的全日制本科考生。

考生群体	实践实考人数	实践通过人数	实践通过率（%）	综合考试实考人数	综合考试通过人数	综合考试通过率（%）	总通过率（%）
本校	419	402	95.94	401	337	84.04	80.43
全国	68672	61741	89.91	60831	48351	79.48	70.41

*注：综合考试即医学综合考试。

3.3 学科掌握情况

3.3.1 基础医学

表 3-3 基础医学各学科掌握情况

学科	考生群体	平均分	标准差	最高分	最低分	掌握率（%）
病理生理学	本校	4.15	1.36	7	0	51.88
	辖区内学校	4.22	1.42	8	0	52.75
	全国	4.46	1.50	8	0	55.75
病理学	本校	7.36	1.89	12	0	52.57
	辖区内学校	7.38	1.87	13	0	52.71
	全国	7.66	2.04	15	0	54.71
解剖学	本校	4.09	1.32	7	0	51.13
	辖区内学校	3.92	1.31	8	0	49.00
	全国	4.07	1.38	8	0	50.88
生理学	本校	8.81	1.98	14	0	62.93
	辖区内学校	8.77	2.00	14	0	62.64
	全国	9.16	2.13	14	0	65.43
生物化学	本校	5.76	2.08	11	0	52.36
	辖区内学校	5.89	2.06	11	0	53.55
	全国	6.53	2.15	11	0	59.36
药理学	本校	4.53	1.41	8	1	56.63
	辖区内学校	4.69	1.42	8	0	58.63
	全国	4.71	1.51	8	0	58.88
医学免疫学	本校	1.92	1.10	5	0	32.00
	辖区内学校	2.16	1.13	6	0	36.00
	全国	2.24	1.18	7	0	37.33
医学微生物学	本校	3.34	0.87	5	0	55.67
	辖区内学校	3.41	0.96	6	0	56.83
	全国	3.35	1.01	7	0	55.83

2023年医师资格考试
成绩分析报告

（桂林医学院，临床执业医师）

国家医学考试中心

第二部分

本校全日制应届本科考生

考试情况比较

报告对象：桂林医学院2022年毕业并参加2023年医师资格考试的全日制本科考生；
全国2022年毕业并参加2023年医师资格考试的全日制本科考生。

考生群体	实践实考人数	实践通过人数	实践通过率（%）	综合考试实考人数	综合考试通过人数	综合考试通过率（%）	总通过率（%）
本校	484	457	94.42	455	358	78.68	73.97
全国	67669	61302	90.59	60698	45064	74.24	66.59

*注：综合考试即医学综合考试。

3.3 学科掌握情况

3.3.1 基础医学

表3-3 基础医学各学科掌握情况

学科	考生群体	平均分	标准差	最高分	最低分	掌握率（%）
病理生理学	本校	4.17	1.32	8	0	52.13
	辖区内学校	4.18	1.33	8	0	52.25
	全国	4.22	1.36	8	0	52.75
病理学	本校	9.46	1.95	14	4	67.57
	辖区内学校	9.47	1.91	14	2	67.64
	全国	9.38	2.05	14	0	67.00
解剖学	本校	5.22	1.41	9	2	58.00
	辖区内学校	5.17	1.43	9	1	57.44
	全国	4.95	1.51	9	0	55.00
生理学	本校	7.86	1.72	12	2	60.46
	辖区内学校	7.86	1.66	13	2	60.46
	全国	8.00	1.80	13	0	61.54
生物化学	本校	5.56	1.78	10	1	55.60
	辖区内学校	5.88	1.73	10	1	58.80
	全国	6.10	1.90	10	0	61.00
药理学	本校	4.15	1.48	7	0	51.88
	辖区内学校	4.01	1.46	8	0	50.13
	全国	4.05	1.45	8	0	50.63
医学免疫学	本校	3.56	1.25	6	0	59.33
	辖区内学校	3.83	1.25	6	0	63.83
	全国	3.82	1.28	6	0	63.67
医学微生物学	本校	3.01	1.06	6	0	50.17
	辖区内学校	3.18	1.04	6	0	53.00
	全国	3.04	1.09	6	0	50.67

2024年医师资格考试成绩分析报告

桂林医学院

临床执业医师

国家医学考试中心

第二部分

全日制应届本科考生考试情况

报告对象：

桂林医学院的全日制应届本科考生（以下简称本校考生）。

全国/辖区内学校/双一流高校/原211高校/原985高校/总通过率最高学校的全日制应届本科考生。

应届本科考生即2023年毕业并参加2024年医师资格考试的本科考生。

辖区内学校的筛选条件：

在临床执业医师考试中，学校所属的省（或直辖市/自治区）内，实践技能考试实考人数大于等于10人的院校。

表2-2 不同群体考生医学综合考试情况

考生群体	综合考试实考人数	综合考试通过人数	综合考试通过率（%）	综合考试通过率所处等级
本校	591	525	88.83	B
全国	65093	53825	82.69	－－
辖区内学校	2559	2251	87.96	B
双一流高校	9422	8520	90.43	B
原985高校	4154	3957	95.26	A
原211高校	7866	7051	89.64	B
总通过率最高学校	22	22	100.00	A

注：医学综合考试通过率是指医学综合考试通过人数占医学综合考试实考人数的百分比。

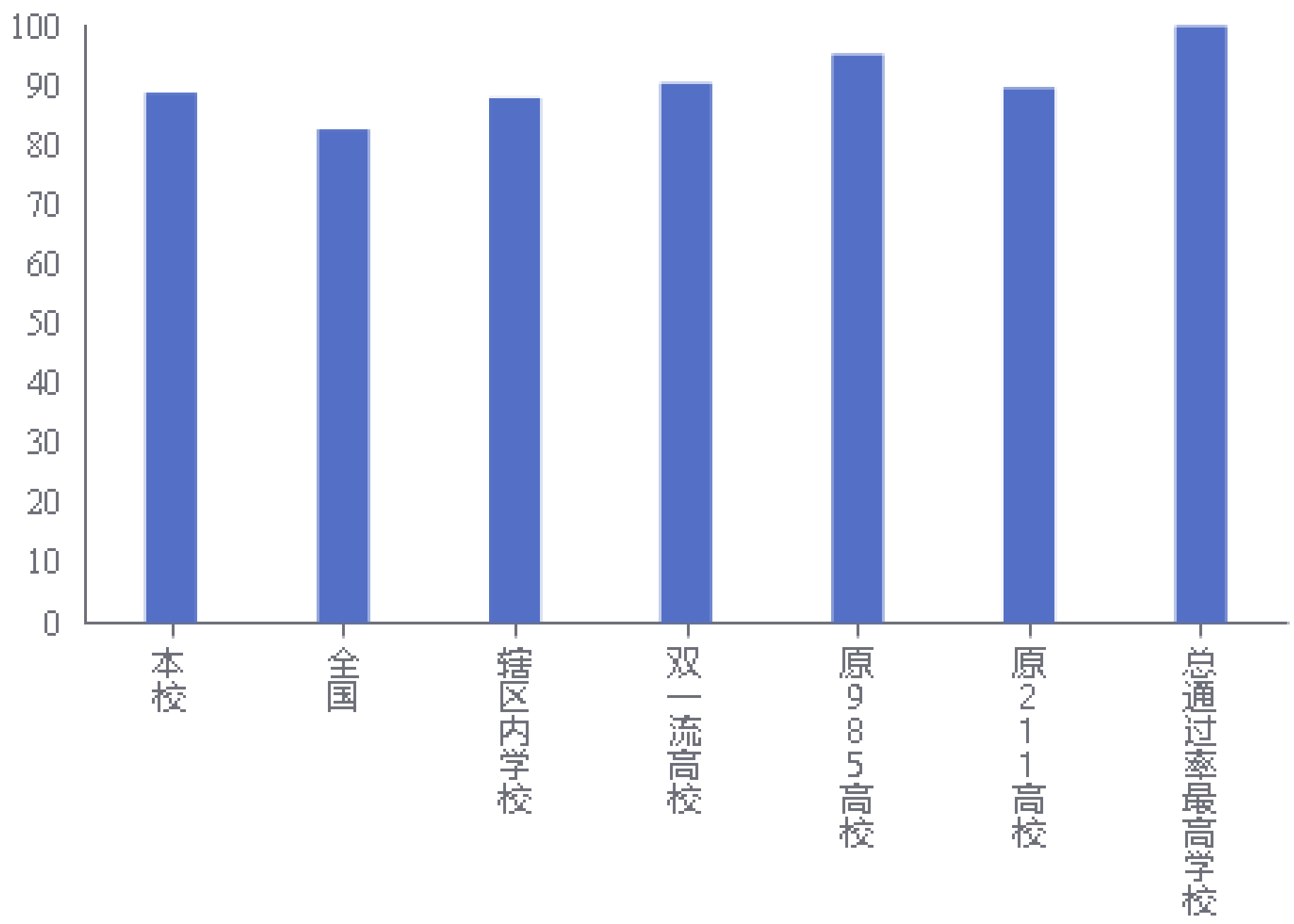

图2-2 不同群体考生医学综合考试通过率（%）

3.3 专业知识点掌握情况

3.3.1 基础医学专业知识点

表3-3 基础医学专业知识点掌握率（%）

专业	本校	全国	辖区内学校	双一流高校	原985高校	原211高校	总通过率最高学校
生物化学	64.65	66.31	63.05	69.05	69.60	68.68	70.91
生理学	58.95	60.18	57.60	63.09	63.54	62.77	62.94
病理学	70.66	70.29	70.79	73.64	75.39	73.32	77.92
病理生理学	74.11	72.78	73.17	76.67	78.15	76.17	82.39
解剖学	30.52	28.30	31.56	32.37	35.16	32.58	36.36
药理学	57.19	54.27	56.31	55.04	56.63	54.65	53.25
医学免疫学	51.86	52.45	53.75	57.79	60.76	57.88	64.39
医学微生物学	59.90	61.62	61.57	67.32	70.33	67.52	68.18

注：1. 同一试题可能包含多个专业知识点。

2. 掌握率是指每个专业知识点考生的平均分占该专业知识点满分的百分比，掌握率的取值范围在0%-100%（下同）。

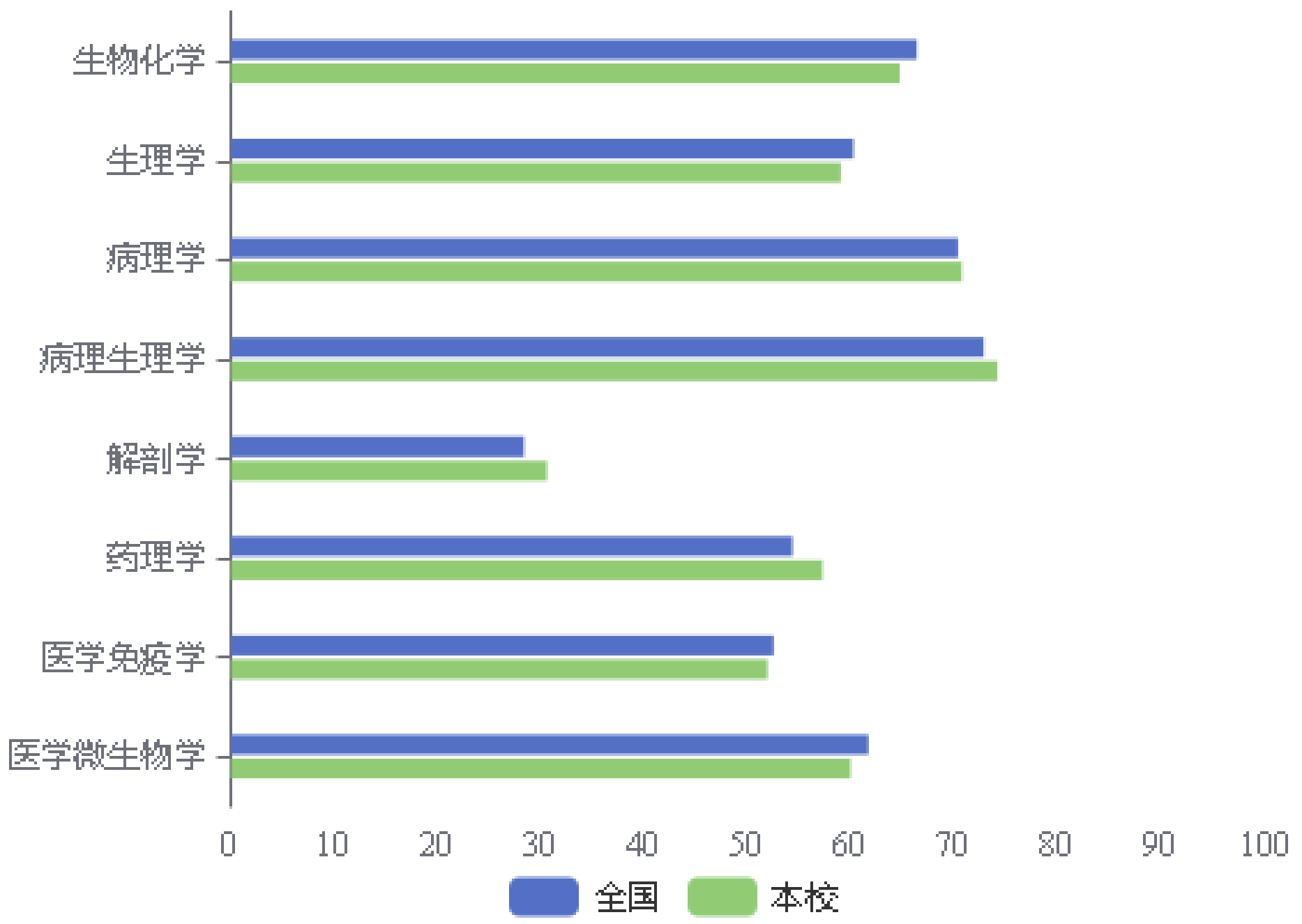

图3-8 基础医学专业知识点掌握率（%）

2025年医师资格考试

成绩分析报告

桂林医科大学

临床执业医师

国家医学考试中心

第二部分

全日制应届本科考生考试情况

报告对象：

桂林医科大学的全日制应届本科考生（以下简称本校考生）。

全国/辖区内学校/双一流高校/原211高校/原985高校/总通过率最高学校的全日制应届本科考生。

应届本科考生即2024年毕业并参加2025年医师资格考试的本科考生。

辖区内学校的筛选条件：

在临床执业医师考试中，学校所属的省（或直辖市/自治区）内，实践技能考试实考人数大于等于10人的院校。

表2-2 不同群体考生医学综合考试情况

考生群体	综合考试实考人数	综合考试通过人数	综合考试通过率（%）	综合考试通过率所处等级
本校	681	575	84.43	C
全国	65698	54085	82.32	--
辖区内学校	2874	2489	86.60	C
双一流高校	10046	9090	90.48	B
原985高校	4167	3939	94.53	A
原211高校	7774	6957	89.49	B
总通过率最高学校	52	52	100.00	A

注：医学综合考试通过率是指医学综合考试通过人数占医学综合考试实考人数的百分比。

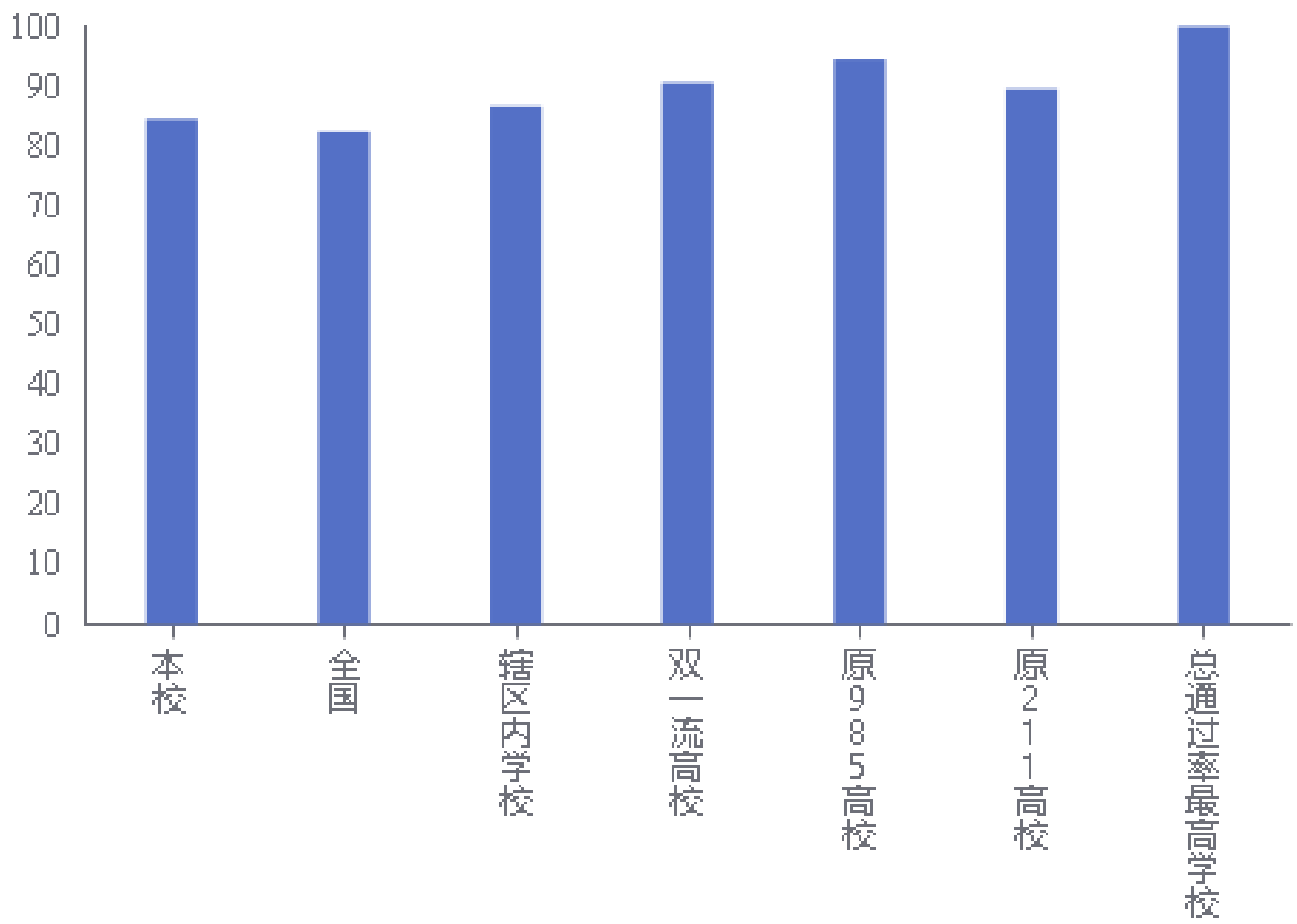

图2-2 不同群体考生医学综合考试通过率（%）

3.3 专业知识点掌握情况

3.3.1 基础医学专业知识点

表3-3 基础医学专业知识点掌握率（%）

专业	本校	全国	辖区内学校	双一流高校	原985高校	原211高校	总通过率最高学校
生物化学	58.94	62.16	58.94	66.08	67.44	65.92	70.96
生理学	61.89	65.67	62.28	66.73	65.59	65.66	65.53
病理学	66.28	69.46	67.43	71.52	71.98	70.65	70.47
病理生理学	76.47	76.07	76.41	79.73	80.49	79.05	84.38
解剖学	55.93	50.24	52.69	56.65	59.35	56.21	59.62
药理学	62.93	64.33	63.41	67.88	69.19	67.26	69.23
医学免疫学	40.60	46.11	44.74	55.96	59.89	55.66	60.26
医学微生物学	57.25	59.20	61.38	62.43	64.43	62.22	66.35

注：1. 同一试题可能包含多个专业知识点。

2. 掌握率是指每个专业知识点考生的平均分占该专业知识点满分的百分比，掌握率的取值范围在0%-100%（下同）。

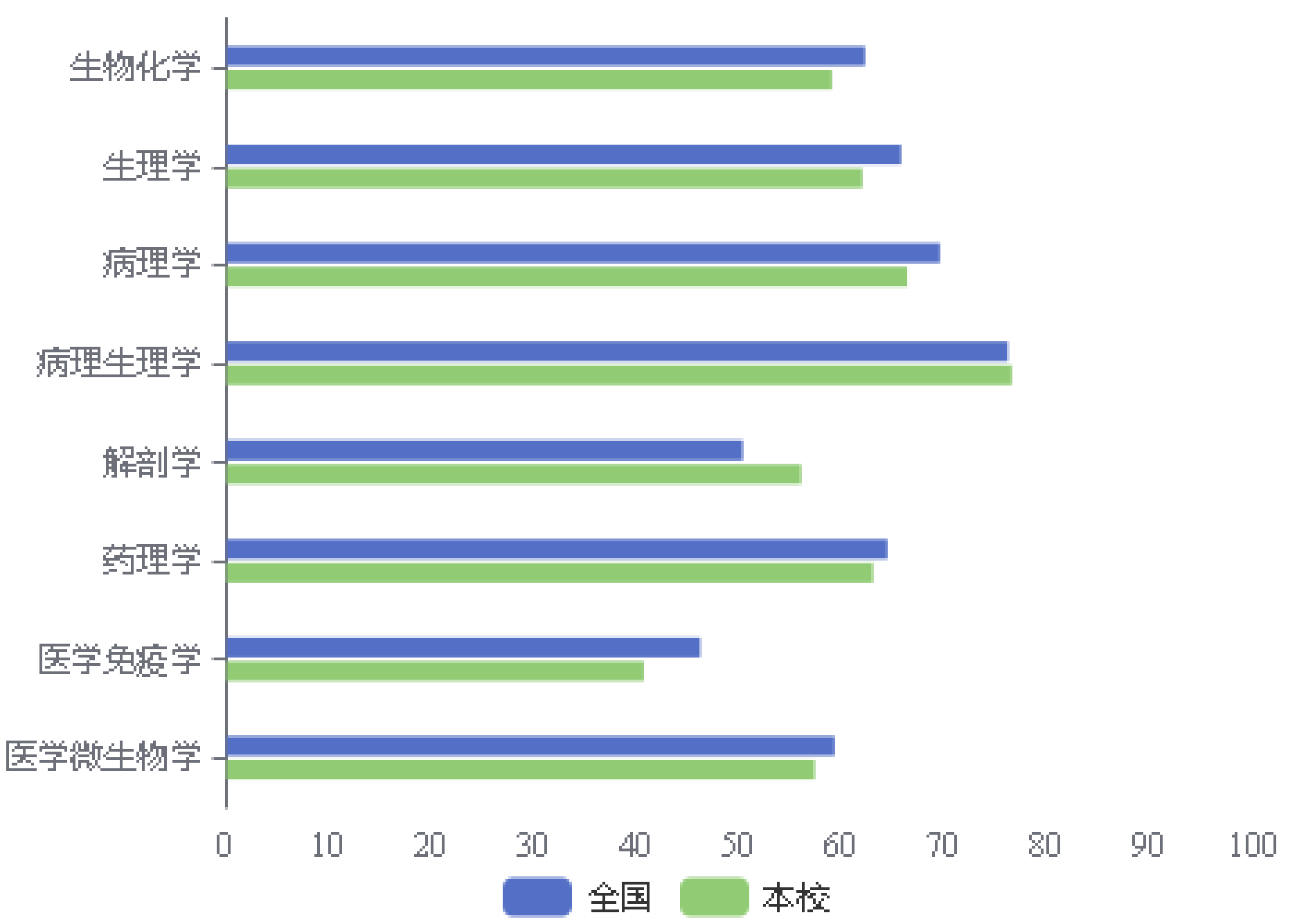

图3-8 基础医学专业知识点掌握率（%）
